# Barriers and facilitators to implementation of healthy food and drink policies in public sector workplaces: a systematic literature review

**DOI:** 10.1093/nutrit/nuad062

**Published:** 2023-06-19

**Authors:** Magda Rosin, Sally Mackay, Sarah Gerritsen, Lisa Te Morenga, Gareth Terry, Cliona Ni Mhurchu

**Affiliations:** School of Population Health, The University of Auckland, Auckland, New Zealand; School of Population Health, The University of Auckland, Auckland, New Zealand; School of Population Health, The University of Auckland, Auckland, New Zealand; Research Centre for Hauora and Health, Massey University, Wellington, New Zealand; School of Clinical Sciences, Auckland University of Technology, Auckland, New Zealand; National Institute for Health Innovation, The University of Auckland, Auckland, New Zealand

**Keywords:** barrier, challenge, enabler, facilitator, food environment, healthy food policy, implementation, workplace

## Abstract

**Context:**

Many countries and institutions have adopted policies to promote healthier food and drink availability in various settings, including public sector workplaces.

**Objective:**

The objective of this review was to systematically synthesize evidence on barriers and facilitators to implementation of and compliance with healthy food and drink policies aimed at the general adult population in public sector workplaces.

**Data Sources:**

Nine scientific databases, 9 grey literature sources, and government websites in key English-speaking countries along with reference lists.

**Data Extraction:**

All identified records (N = 8559) were assessed for eligibility. Studies reporting on barriers and facilitators were included irrespective of study design and methods used but were excluded if they were published before 2000 or in a non-English language.

**Data Analysis:**

Forty-one studies were eligible for inclusion, mainly from Australia, the United States, and Canada. The most common workplace settings were healthcare facilities, sports and recreation centers, and government agencies. Interviews and surveys were the predominant methods of data collection. Methodological aspects were assessed with the Critical Appraisal Skills Program Qualitative Studies Checklist. Generally, there was poor reporting of data collection and analysis methods. Thematic synthesis identified 4 themes: (1) a ratified policy as the foundation of a successful implementation plan; (2) food providers’ acceptance of implementation is rooted in positive stakeholder relationships, recognizing opportunities, and taking ownership; (3) creating customer demand for healthier options may relieve tension between policy objectives and business goals; and (4) food supply may limit the ability of food providers to implement the policy.

**Conclusions:**

Findings suggest that although vendors encounter challenges, there are also factors that support healthy food and drink policy implementation in public sector workplaces. Understanding barriers and facilitators to successful policy implementation will significantly benefit stakeholders interested or engaging in healthy food and drink policy development and implementation.

**Systematic Review Registration:**

PROSPERO registration no. CRD42021246340.

## INTRODUCTION

Diet-related diseases, such as cardiovascular disease, type 2 diabetes, and cancer, contribute to the high burden of noncommunicable diseases globally.^1^ In 2017, 11 million (22%) of all adult deaths and 255 million (15%) of all adult disability-adjusted life-years were attributable to dietary risks factors, mainly high sodium and diets low in whole grains, fruits, nuts, seeds, vegetables, and fiber.^2^ Unhealthy eating patterns, together with sedentary lifestyles, have been identified as the main causative agents of obesity,^3^ affecting populations in both high-income and low- to middle-income countries^4^ and contributing to an increase in social inequalities.^5^ This unhelpfully frames obesity as a problem relating to individual choice; therefore, health nutrition policy may not be viewed as a legitimate public health priority. In fact, healthy eating can positively affect psychological well-being^6^ and overall health^7^ regardless of weight status.^8^^,^^9^ Therefore, interventions to improve population nutrition will have a substantive public benefit. Given that what people choose to eat is largely socially determined^10–12^—that is, based on what they can afford, what is available and what they know—effective public health interventions must consider food environments, because research has shown that dietary interventions targeted only at the individual level generally produce only small and temporary changes in health outcomes.^2^^,^^13^

In 2021, the World Health Organization (WHO) published an action framework for the implementation of effective public healthy food policies aimed at increasing the provision of food and drink that contribute to healthy diets while reducing the provision of unhealthy equivalents.^14^ With the majority of the adult population working^15^^,^^16^ and spending about a quarter to half of their waking hours in a workplace,^15^ workplaces have been recognized as an important environment for the implementation of food policies promoting health and well-being of employees.^14^^,^^17^ Cafeterias, retail food outlets, and vending machines located in workplaces represent important sources of at least some of the food and snacks consumed at work.^17^^,^^18^ According to a 2019 Organization for Economic Cooperation and Development (OECD) report,^5^ private sector workplace-based interventions to improve diet may be common, although they may be problematic to identify and monitor. Some countries, states, and districts have introduced healthy food and drink standards in public sector workplaces; however, there have been few comprehensive, broad, and regular evaluations of these policies to date.^19^

## RATIONALE FOR THE REVIEW

Food vendors and caterers may face barriers in the provision of healthy and nutritious food^20^ and, by extension, in the implementation of healthy food environment policies. Overall, the evidence base lacks a synthesis of the barriers and facilitators to implementation and compliance with healthy food environmental workplace policies, representing an important gap in the workplace health promotion area. The findings of this comprehensive review will benefit any stakeholder with an interest in, or that engages in, healthy food and drink workplace policy development (eg, local or national governments), adoption, implementation or compliance, by providing a deeper understanding of barriers, facilitators, and other unintended or unforeseen outcomes of healthy food and drink policy implementation in public sector workplaces. Ultimately, the findings of this review may help mitigate negative perceptions of food environment policy implementation, as well as identify factors that are beneficial or essential for successful policy implementation, which may prove cost- and resource-saving for policy makers and public sector organizations. The primary aim of this review was to identify the barriers and facilitators to implementation of and compliance with healthy food and drink policies aimed at the general adult population in public sector workplaces.

## METHODS

The systematic review methods were informed by the Cochrane Qualitative and Implementation Methods Group guidance^21^^,^^22^ and the Preferred Reporting Items for Systematic Reviews and Meta-Analysis reporting (PRISMA) 2020 statement.^23^^,^^24^ See Appendix S1 in the Supporting Information online for detailed PRISMA checklist. The protocol was registered on Prospero (registration no. CRD42021246340), with no major changes made to the original protocol.

### Search strategy and information sources

The search strategy for published literature linked together 4 key concepts: “workplace,” “food,” “food environment” and “policy” with the AND operator and was primarily tailored for the MEDLINE database using subject headings and then adapted for other databases (Embase, Scopus, CINAHL, Cochrane CENTRAL, APA PsycINFO, and PubMed). Grey literature was searched using ProQuest Dissertations and Theses Global, OpenGrey, Grey Literature Reports, Eldis, BASE, WHO Institutional Repository for Information Sharing (IRIS), International Food Policy Research Institute (IFPRI), the NOURISHING database, OECD iLibrary, and Google Scholar (first 200 references), and related reviews using Epistemonikos. All databases were searched on April 7–8, 2021. Websites of government agencies and nongovernmental organizations were searched in key English-speaking countries for any relevant reports. Additionally, a search update alert was set up for the MEDLINE, Embase, CINAHL and Scopus databases up to and including July 5, 2021. Backward citation hand-searching was conducted on all included publications and relevant systematic reviews, and the Google Scholar forward citation search function was used on all included publications. The publication date for studies was limited to the year 2000 and later, reflecting the time WHO recognized the importance of environmental factors in improving health outcomes.^25^ Full details of search log and results can be found in Appendix S2 in the Supporting Information online.

### Screening and selection of studies

**Figure 1:**
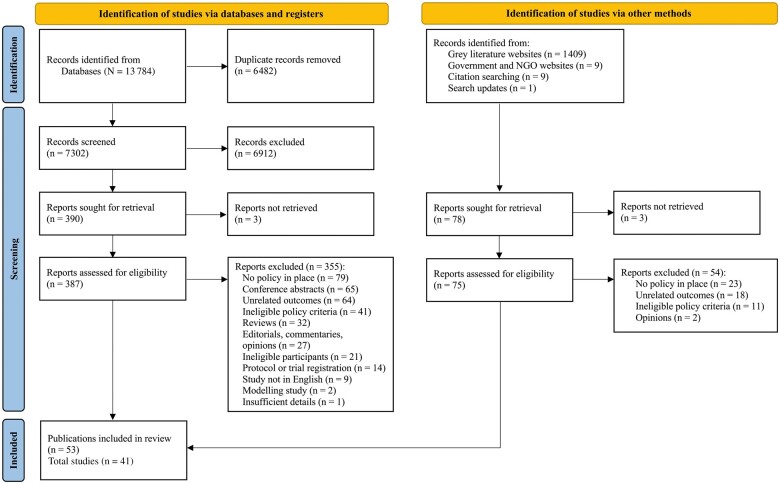
**Study selection flow diagram with accordance to 2020 PRISMA guidelines**.^24^*Abbreviation:* NGO, nongovernmental organization.

All identified publications were exported into Zotero, version 5.0.92, reference management software and duplicates were removed manually. Grey literature records were prescreened for relevance by M.R. Two reviewers (M.R. and S.M.) independently screened and selected eligible studies in a standardized blind manner using Covidence systematic review software.^26^ At the title and abstract screening stage, clearly irrelevant records were excluded. For the remaining records, full-text versions were obtained and assessed for eligibility against PICOS criteria (Table 1). Determining policies’ eligibility involved searching for the policy online to obtain more information where necessary. A third team member (C.N.M.) was consulted to seek clarification and guidance on the definition of healthy food and drink policies, resulting in more precise alignment with the WHO definition.^14^ Any discrepancies in screening and selection results between the 2 reviewers were discussed and a consensus reached. All studies excluded from the review at the full-text stage and the main exclusion reasons were noted. Results from the same study using the same participants and methods but presented as separate reports were collated to represent a single study for this review. Studies were not excluded on the basis of the methodological limitations’ assessment, although study quality-assessment results were used to assess the confidence in the synthesis findings. A PRISMA flow diagram (Figure 1)^24^ illustrates the search results and study selection process.

**Table 1:** PICOS criteria for inclusion of studies

	Inclusion criteria	Exclusion criteria
Participants	Supply-side stakeholders involved directly in the implementation of healthy food and drink policies, including food service and retail managers and staff; caterers and catering staff; head cooks and kitchen staff preparing food and drink; staff providing, supplying, and serving food and drink; organizational dietitians, nutritionists; workplace representatives, administrators and support staff Settings (any geographic location): public sector workplaces: public health care facilities (eg, hospitals, clinics), government agencies, local councils, public universities, and public sports clubs; food environments: staff cafeterias, retail food outlets, vending machines, catering, and fundraising offered on the premises or on behalf of public sector workplaces	Demand-side stakeholders: consumers of foods and drinks (staff, visitors, and students in public sector workplaces) Settings: schools, early childhood education centers (preschools and kindergartens), tertiary residential education settings (eg, student dining halls), private sector workplaces, private universities, retail settings (eg, supermarkets), any food retail outlets outside the public sector workplace setting (eg, restaurants, takeaways), settings with institutionalized or clinical populations (eg, prisons, aged care, in-patient meal service)
Intervention	Healthy food and/or drink policy targeting food and drink available to general adult population (staff and visitors) in public sector workplaces Alternative terms for *policy*: procurement, supply, guideline, strategy, standard, criteria, regulation, program, and initiative	Healthy food and/or drink policy specifically aimed at children and youth only Labelling or pricing policies only (ie, those without associated nutrient criteria) Policies focused predominantly on breastfeeding; health systems (eg, patient waiting time, vaccination); communicable diseases (eg, malaria, HIV); undernutrition (eg, fortification, supplementary foods); food insecurity; alcohol, tobacco; accidents; physical activity; food safety and functional food topics (eg, allergens, food additives, genetically modified foods); agriculture (eg, crop yield, pesticide); environmental issues (e.g., greenhouse gases, food waste)
Comparison	Studies with or without comparison group	Nil
Outcomes	Primary outcomes: any reported barriers or facilitators to implementation of and compliance with public sector workplace food environment policies (after adoption of the policy) Secondary outcomes: any unintended or unforeseen consequences of the adoption, implementation of, and compliance with public sector workplace’s food environment policies	Potential (ie, not yet experienced) barriers and facilitators to healthy food and drink policies implementation
Study design	All study designs (including grey literature publications) English language	Editorials, commentaries, and opinion pieces Non-English language

### Criteria for considering studies for this synthesis

The WHO definition of a healthy food and drink policy (ie, a policy that “establishes nutrition criteria to increase the availability of foods and beverages that promote healthy diets, and/or limit or prohibit the availability of foods and beverages that contribute to unhealthy diets”^14^) was used to determine whether a policy met the inclusion criteria. Nutrition criteria encompassed any set of nutrient-based (eg, sodium, saturated fat) or food-based (eg, meals, snacks) standards specifying what food and drinks were allowed or recommended to be sold or served on the premises or on behalf of the public sector workplaces.^14^ Additionally, criteria may have referred to preparation or cooking methods or further display, availability, or promotion restrictions for food or drink.^14^

The participants of interest included any supply-side stakeholder involved directly in implementation of healthy food and/or drink policy in public sector workplaces (eg, workplace food providers, organizational dietitians), with no restrictions on the age of participants. Studies reporting solely on data collected from demand-side stakeholders (eg, workplace staff or visitors) were excluded because, as consumers of food and drinks, they are generally not involved in healthy food and drink policy implementation, although it is likely that their preferences and purchasing practices influence supply-side stakeholders’ implementation efforts. However, it was expected that the supply-side stakeholders would report these factors should they be influential in implementing food policies in public sector workplaces. Studies reporting on data collected from both supply- and demand-side stakeholders were included providing data specific to supply-side stakeholders could be extracted separately.

There was no restriction on the location of the study. However, to be included in the review, studies had to be published in English. Scoping searches suggested relevant data were collected mainly using qualitative methods, and the results included participant quotes and descriptive themes and categories. A pragmatic decision was made to exclude studies published in languages other than English, because translation into English might have compromised the original intended meaning.

### Extraction and synthesis of contextual and methodological data

The Joanna Briggs Institute Qualitative Data Extraction Tool^27^ and Cochrane guidance^22^ were used to extract contextual and methodological study characteristics, with focus on the elements of most relevance for this review (eg, data on demand-side stakeholders were not extracted). One reviewer (M.R.) collated information on the study or policy name with associated publications (author[s] and year), geographic location, aim(s), policy (type, approach, year), study settings (eg, hospital, council building), and the number of sites, participants and sample size(s), study design (eg, qualitative, quantitative, mixed methods) and methods used (eg, interviews, surveys), and data analysis approach (eg, theoretical models used). Where details were not available in the full text or supplementary records, they were indicated as “not reported.” Also noted was whether the study conducted food environment audits, customer surveys, and sales data analysis, all of which are common in studies evaluating the implementation and impact of healthy food and drink policies. Another reviewer (S.G.) cross-checked a random subsample of studies (50%) and recommended minor changes, mainly inclusion of the number of study sites, which were added for the remaining studies.

### Thematic synthesis of findings

The full text and supplementary materials of the included studies were reviewed by 1 author (M.R.) to extract data verbatim (as provided by studies’ authors, including any quantitative data relevant to the review’s research question) from findings, discussion, and conclusion sections. For studies that also addressed private workplaces or included additional noneligible policies, where possible, the findings were extracted for only those workplaces and policies that met the inclusion criteria. Data included, but were not limited to, qualitative themes and sub-themes; supporting quotations, explanations, interpretations, conclusions, lessons learned, and observational excerpts; and any relevant tables, figures, and logic models. Key findings from each included study are presented in Appendix S3 in the Supporting Information online. All extracted data were copied into Microsoft Word documents and uploaded and analyzed with assistance of QSR's NVivo software for qualitative data analysis.^28^ Analysis and synthesis of findings drew on thematic synthesis as described by Thomas and Harden^29^ because it is considered by the Cochrane guidelines^22^ to be the most suitable method for undertaking a qualitative evidence synthesis, offering analytic flexibility with regard to different formats of data presentation. No a priori framework was used in the analysis. Rather, coding and theme development followed a research question–led, reflexive thematic analysis approach as outlined by Braun and Clarke^30^ and modified for this review.

Thematic synthesis was based on a partially overlapping 6-stage process.^29^^,^^30^ First, preceding review and multiple readings enabled 1 reviewer (M.R.) familiarization with and immersion in the data. Second, data coding was carried out the same reviewer and the codes were shared and refined with another reviewer (G.T.) experienced in the reflexive thematic analysis approach. Next, 1 author (M.R.) inductively coded all verbatim data from the primary studies, regardless of whether they directly addressed the review's questions or outcomes. Although the views of demand-side stakeholders and results of any food environment audits or sales data assessment were of no direct interest in this review, they were still coded to provide support for the final analytical themes. However, these codes were clearly distinguished from the views and opinions of the supply-side stakeholders. Each sentence or extract was allowed to fit into at least 1 code. No hierarchical code structure was developed, that is, the created codes were “free,” and represented a mixture of semantic (descriptive) and latent (interpretative) codes so as to not prematurely create patterns or themes without realizing the full potential of the data. After initial coding, 1 author (M.R.) reviewed the codes and continuously revised them as the coding and analysis proceeded. The text in all assigned codes was also examined for consistency of interpretation, and a pool of generated codes was shared with all authors. Third, all reviewers independently familiarized themselves with the generated codes and attempted to cluster the codes into initial (candidate) themes using an online software tool (miro.com). Subsequently, all authors met and together discussed their impressions and initial themes. Descriptive themes and sub-themes (encompassing key interconnected concepts within a theme) were developed from the codes, initial themes, and group discussion, resulting in a draft summary of the findings across the studies that was used in stage 4. Fourth, descriptive themes were further analyzed to generate ideas, explanations, and hypotheses (including those related to the review's questions), which were agreed upon and finalized through discussion by all review authors. Fifth, detailed analysis and informative naming of the themes in this review enabled refining of each analytical theme definition, focus, and scope to address the review’s research question by synthesizing and interpreting the findings of the primary studies to generate new concepts. Sixth, the analytical description of each theme was supported by data extracts from the included studies.

### Assessment of the methodological strengths and limitations of studies

The quality of all included studies was appraised by 1 author (M.R.) using the Critical Appraisal Skills Program (CASP) tool for qualitative studies,^31^ commonly used in WHO and Cochrane guidelines,^22^ even though they were methodologically quite distinctive. Assessment was guided by the 10 questions and their associated prompts provided in the CASP tool^31^ (with question 2 modified to include all, rather than solely, qualitative methodologies). For studies with aims and methods other than those addressing the review’s research question, the focus of the appraisal was purely on the components and methods that were used to address the barriers and facilitators to healthy food and drink policy implementation. Studies were not treated as of lower quality, because a quantitative or mixed-methods design was used. A purposively selected subsample of studies (25%), representing a range of methodological limitations results from the appraisal, were cross-checked for consistency by another reviewer familiar with qualitative methods (G.T.). The overall methodological limitations were recorded on a scale ranging from negligible to minor, moderate, or major limitations.

## RESULTS

### Search and selection results

Initial scientific databases and Google Scholar searches resulted in 13 784, deduplicated to 7302 publications and imported into Covidence for eligibility assessment. At the abstract and screening stage, 6912 records were deemed irrelevant. Full texts of the remaining 390 records were searched but could not be located for 3 studies, and the reviewers were unable to contact the study authors because no corresponding addresses were provided in the publications. Full-text review of the remaining 387 records resulted in 32 eligible publications included in this review. Grey literature database searches generated 1409 records, which were deduplicated to 1238 records. Prescreening excluded 1179 records, leaving 59 records for full-text review, of which 1 thesis could not be retrieved because it was under embargo and 2 publications could not be retrieved (n = 56 records remained). Additionally, 19 records were identified via searches of government and nongovernmental organization websites, citation searches, and automated search updates. Full-text review of the 75 grey literature records resulted in 21 eligible publications. Together, 53 publications^32–84^ were eligible for inclusion. Individual publications reporting on the same study and participants were collated to represent a single study as the unit of interest in this review, resulting in 41 relevant studies (see Figure 1).

### Contextual and methodological characteristics of included studies

Characteristics of the 41 included studies are summarized in Table 2.^32–84^ The majority of the studies were conducted in Australia (n = 11),^32–47^ the United States (n = 11),^48–62^ and Canada (n = 9).^63–71^ The remaining studies were conducted in South Korea (n = 2),^72^^,^^73^ the Netherlands (n = 2),^74^^,^^75^ and 1 each in Scotland,^76^^,^^77^ Wales,^78^ England,^79^ Switzerland,^80–82^ Denmark,^83^ and New Zealand.^84^ Studies were either carried out in a single type of facility or included more than 1 type of public workplace facility, namely, healthcare facilities (n = 22), recreation and sports centers (n = 12), federal or local council government buildings (n = 9), public universities (n = 2), army facilities (n = 2), and a police department (n = 1). Two studies did not specify the type of workplace facilities.^72^^,^^84^ Six studies were conducted in a mix of public sector and private companies.^55^^,^^73^^,^^74^^,^^80–84^ The number of sites ranged from 1^46^^,^^49^^,^^69^ to 278 sites.^43^^,^^44^

**Table 2:** Contextual and methodological characteristics of included studies

Study, reference(s), location	Study aims	Policy (type, approach, year)^a^	Study settings (no. of sites)	Participants (sample size)	Study design and methods	Data analysis approach^b^
Healthy Choices in Victorian Recreation Centres Blake et al (2021)^32^ Melbourne, Victoria, Australia	To investigate changes in stakeholder commitment and perceptions of the purpose, challenges, and benefits of healthy food and beverage provision in community sports settings over time during the stepwise implementation of a healthy beverage policy and to explore sources of heterogeneity across organizations and individuals	Healthy Choices: policy guidelines for sports and recreation centers (voluntary, 2014)	Local-government owned sports and recreation centers (n = 4)	Total (n = 6); sports and recreation center managers (n = 2), council managers (n = 2), dietitian (n = 1), food service manager (n = 1)	Mixed methods,^c^^,^^d^^,^^e^ convergent, parallel Repeat semi-structured interviews, repeat quantitative stakeholder surveys (using Commitment to Organizational Change scale)	Critical realist perspective combining realist ontology with a constructivist epistemology approach; analysis of inductive reflexive thematic interviews; descriptive analysis of the remaining data; side-by-side comparison narrative
Healthy Choices in Alfred Health (Boelsen-Robinson et al, 2017)^34^; Peeters et al (2017)^37^; Boelsen-Robinson (2019)^33^ Victoria, Australia	To understand the context within which the policy was implemented, and to explore factors that may explain the impact of the policy on sales of healthy and unhealthy foods and beverages	Healthy Choices: Food and drink guidelines for Victorian public hospitals (2010)	Alfred Health Metropolitan health service facilities, major hospital sites (n = 3), with focus on 37 vending machines	Total (n = 4); health promotion manager (n = 1), dietitian (n = 1), procurement manager (n = 1), senior executive (n = 1)	Mixed methods^e^ explanatory sequential design Semi-structured in-depth interviews, snowballing sampling until no new themes emerged from the interviews; analysis of sales data was used to inform the qualitative component	Open-ended coding (block and segment approach using thematic analysis)
Healthy Choices in Alfred Health (Boelsen-Robinson et al, 2019)^36^; Victorian Health Promotion Foundation (2017)^38^; Boelsen-Robinson et al (2016)^35^; Boelsen-Robinson (2019)^33^ Victoria, Australia	To evaluate the experience and perspectives of those implementing the healthy-food retail policy within an independent food retailer located on site of a health service, in order to inform the translation of such policies into other organizations	Healthy Choices: Food and drink guidelines for Victorian public hospitals (2010, mandatory)	Alfred Health Metropolitan health service facilities; independently owned retail outlets (n = 5)	Total (n = 7); administrative assistant (n = 1), external supplier (n = 1), food outlet owner (n = 1), head chef (n = 1), health promotion manager (n = 1), dietitian (n = 1), senior executive (n = 1)	Qualitative In-depth, semi-structured face-to-face and telephone interviews, descriptive method enquiry	Theory of social ecology approach; thematic analysis, open-coding (inductive, block and segment approach)
Victorian Healthy Choices and Healthy Catering case studies Chang et al (2016)^39^ Melbourne Inner East Catchment, Victoria, Australia	To explore the implementation of healthy food and drink policies and guidelines in a range of settings across local government areas within Victoria To explore how local initiatives and policy interventions can influence the food environment To support identification of opportunities to change food environment and improve access to healthy food options	Healthy Choices policy guidelines for sports and recreation centers (2014, voluntary); Healthy Choices: food and drink guidelines for Victorian public hospitals (2010, voluntary); Healthy Catering Policy and Guidelines (Knox City Council, 2014)	Sports and recreation centers (n = 1), the Alfred hospital (n = 1), local council building (n = 1)	Total (not reported); key individuals involved in each initiative (mostly highly placed health promotion officers and program managers)	Series of (mixed methods) case studies^c^^,^^e^ Semi-structured interviews, investigation of grey literature	Not reported
Healthy Choices in ACCHOs MacDonald et al (2016)^42^ Victoria, Australia	To describe initiatives leading to increases in healthy catering choices and related challenges for Aboriginal workplace health promotion practice To identify shifts in organizational practice, policy, and attitudes	Healthy Choices: food and drink guidelines for Victorian public hospitals (2010), supported by a pilot project strengthening nutrition policy development and implementation (18 months)	ACCHOs (n = 5)	Total (not reported); managers, project champions, staff	Mixed-methods^d^ pilot project Document analysis (log of implementation steps and challenges); structured interviews and questionnaires	Thematic analysis, comparative analysis of baseline and follow-up data
Victorian policies in sport and recreation facilities Riesenberg et al (2020)^45^ Victoria, Australia	To assess policies, attitudes that inform obesity-prevention practices, and the provision of healthy food and drink options in local government–owned sport and reaction facilities	Various formal healthy food and drink policies in 21 sport and recreational facilities	Local government–owned sport and recreation facilities (n = 49)	Total (n = 49); local governments' managers, coordinators or similar roles	Mixed methods Online cross-sectional survey (closed and open questions)	Descriptive statistics
A Better Choice evaluation Miller et al (2015)^43^; Queensland Health (2010)^44^ Queensland, Australia	To measure the extent of implementation of the strategy in Queensland Health facilities	A Better Choice Healthy Food and Drink Supply Strategy for Queensland Health Facilities (2007, mandatory since 2008)	Government-owned health facilities (n = 278)	Total (n = 158); facility managers (n = 134), volunteer policy district contact officers (food service managers, dietitians, nutritionists) (n = 24)	Mixed methods^d^ Self-reported online survey (closed and open questions); telephone interviews	Statistical analysis (surveys), thematic analysis (interviews)
Queensland children’s hospital study Walker et al (2020)^46^ Brisbane, Queensland, Australia	To determine the viability of the “Healthier Drinks at Healthcare Facilities” strategy to reduce the amount of free sugar available in beverages and influence consumer purchasing patterns To gain an understanding of the views of retail (food outlet) managers regarding strategy implementation	A Better Choice Healthy Food and Drink Supply Strategy for Queensland Health Facilities (2008, mandatory); Healthier Drinks at Healthcare Facilities strategy (2016)	Major tertiary children's hospital (n = 1)	Total (n = 3); retail food outlet managers	Mixed-methods^c^^,^^e^ design; convergent parallel Semi-structured face-to-face interviews	Content analysis
Healthy Options WA Department of Health, evaluation Western Australia Department of Health (2020)^47^ WA, Australia	To evaluate policy implementation To gather information relevant to a forthcoming review of the policy To guide development of policy support resources	Healthy Options WA: Policy for WA Health Services and Facilities (2008, revised 2009 and 2015, mandatory)	Health facilities, metropolitan (n = 12), regional (n = 13)	Total (n = 27); catering and café managers, kiosk managers, volunteer staff, auxiliary coordinators, external contractors, staff-wellness committee members	Mixed methods^d^ Questionnaire (close and open questions), in-depth interviews	Excel software (not further specified)
Healthy Options WA in East Metropolitan Health Service Law et al (2021)^41^ Perth, WA, Australia	To explore food retailers’ experiences of implementing a mandated healthy food procurement policy across an Australian metropolitan health service	Healthy Options WA: Food and Nutrition Policy for WA Health Services and Facilities (2008, updated 2021, mandatory)	East Metropolitan Health Service hospitals (n = 4) with 44 food outlets	Total (n = 12); food retailers, catering staff, vendors, volunteers	Qualitative In-depth semi-structured small group workshops (combination of presentations and group exercises)	Paradigm of social constructivism approach; inductive thematic content analysis, triangulation
South Australian health facilities evaluation Government of South Australia (2012)^40^ South Australia, Australia	To understand barriers and facilitators to the adoption of the policy across SA Health To seek feedback on the implementation process from sites To assess the extent to which the policy has been embedded in the organizational policies and practices	Healthy Food and Drink Choices for Staff and Visitors in South Australian Health Facilities Policy (2009, mandatory)	Health facilities (hospitals, head office, community, and state-wide health service, clinics) (n = 111)	Total (n = 111); individual roles not reported	Mixed methods^d^ Self-reporting template for health facilities	Thematic analysis, descriptive statistics
Army DFACs intervention Armstrong et al (2021)^48^ Fort Bragg, North Carolina, USA	To gain a better understanding of food-service staff experience and common barriers to the implementation and sustainment of DFAC-based nutrition interventions at 2 US Army DFACs	Special Operations Forces Human Performance Program DFAC-based nutrition intervention (nutrient standards, food choice architecture, labelling)	Army DFACs (n = 2)	Total (n = 168); nonsupervisory civilian contract staff (n = 76), Army food-service–trained soldiers (n = 92)	Qualitative Focus groups (in person) using semi-structured interview guide (on 3 separate occasions during intervention implementation)	Phenomenological approach; iterative thematic content analysis (codebook)
Hubert Building Cafeteria Guidelines Bayne et al (2012)^49^ Washington, DC, USA	To describe vendors’ perspectives on the Health and Sustainability Guidelines and their ability to implement these guidelines in current and future cafeteria contracts	Health and Sustainability Guidelines for Federal Concessions and Vending Operations (2011)	The Department of Health and Human Services (HHS) Hubert H. Humphrey Building Cafeteria (n = 1)	Total (n = 18); federal staff involved in guideline's development and implementation (n = 9), food service vendors' representatives: president (n = 1), vice president (n = 3), communications director (n = 1), wellness director (n = 1), corporate chef (n = 1), purchasing manager (n = 1), consultant providing support (n = 1)	Mixed-methods case study Document review (Health and Sustainability Guidelines); semi-structured telephone interviews	Traditional methods of qualitative data analysis, based on the discernment of themes and patterns in the data through a content analysis
Boston Sodium Reduction Initiative Brooks et al (2017)^50^ Boston, MA, USA	To evaluate a community-level sodium-reduction intervention in Boston, MA To better understand factors influencing intervention implementation	Boston Public Health Commission initiative (2013, focus on tailored sodium reduction in prepackaged items aligning with the Massachusetts School Nutrition Standards for Competitive Foods and Beverages)	Hospitals (n = 7), YMCAs (n = 8), community health centers (n = 4), organizations serving homeless populations (n = 2)	Total (not reported); implementation leaders (directors or staff in the food and nutrition services department) in 21 community institutions	Mixed methods^d^ quasi-experimental pre–post design with no control group Interviews	Not reported
Vidant Healthy Food Environment Policy Gaskins et al (2013)^51^ Greenville, NC, USA	To share the history of how a hospital created a healthy food environment in the absence of national policy, in order to help others achieve an affordable, accessible, and healthy food environment	Vidant Healthy Food Environment Policy (2012)	Hospitals and healthcare facilities (n =10)	Total (not reported); authors include dietitians and employees of health care organization and food service operation	Not reported Not reported	Not reported
Healthy vending policies in US cities Green et al (2020)^52^ Four US cities (2 large, 2 smaller)	To examine factors that facilitated or hindered the implementation of healthy vending policies and initiatives in 4 cities	Various vending nutrition guidelines based on Healthy Workplace Food and Beverage Toolkit (American Heart Association), Alliance for a Healthier Generation, FitPick, National Alliance for Nutrition and Activity, Health and Human Service, Healthy vending policies in other cities, and dietitian’s expertise	Publicly accessible vending machines (n = 8) typically in recreation centers	Total (n = 25); health department staff, staff from the sites where vending machines were located, policy makers, and vendors	Mixed-methods^d^, multiple case study design Semi-structure interviews; documents' review (policies, vendor contracts, nutrition standards)	Interviews; approach not reported Documents; evaluated using checklist (not specified)
US hospital and federal worksites guidelines Jilcott Pitts et al (2016)^53^ United States (various locations)	To examine barriers to and facilitators of implementation, behavioral design strategies used to promote healthier foods and beverages in cafeteria settings, and effects on costs and profits of implementation of the healthier food-service guidelines or initiatives	Health and Sustainability Guidelines for Federal Concessions and Vending Operations (US Department of Health and Human Services, General Services Administration; federal worksites, 2011) Hospital Healthier Food Initiative (Partnership for a Healthier America; hospitals, 2010)	Hospitals (n = 5) and federal worksites (n = 4)	Total (n = 9); hospital (n = 5) and federal worksite (n = 4) food-service operators	Mixed methods Quantitative surveys followed by qualitative, in-depth telephone interviews, purposive sampling	Phenomenological approach; surveys—descriptive statistics; interviews—deductive approach (from survey and interview questions) followed by inductive approach
US Hospital food service guidelines Jilcott Pitts et al (2018)^54^ United States	To examine barriers and facilitators to maintaining financial sustainability of healthy food-service guidelines To synthesize information on best practices for financial sustainability in hospital food service	Nonspecified healthy food service guidelines (eg, Hospital Healthier Food Initiative by Partnership for a Healthier America, 2010)	Hospitals (n = 8)	Total (n = 8); hospital food-service directors	Qualitative In-depth semi-structured interviews with pairs of interviewees with similar operational characteristics; purposive sampling	Deductive from interview guide's headers and inductive; themes consolidated into best practices; participant checking of results
NYC Healthy Hospital Food Initiative Lederer et al (2014)^55^ New York City, NY, USA	To describe the characteristics, sodium- and nutrition-related knowledge, attitudes and behaviors of staff managing cafeterias in New York City hospitals	New York City hospitals Department of Health and Mental Hygiene’s Healthy Hospital Food Initiative (2010, focus on low-sodium offerings)	Public (n = 5) and private not-for-profit (n = 12) hospitals	Total (n = 17); retail and general cafeteria managers (n = 9), assistant directors or directors of food and nutrition services departments (n = 6), executive chef (n = 1)	Quantitative survey In-person surveys (mostly yes/no responses with open ended questions), convenience sampling	Statistical analysis
“Red Apple” Hospitals Project Neffa (2011)^56^ NC, USA	To better understand the factors and motivators the managers and employees responsible for implementing the project considered important To explore how implementers' thoughts about the project's implementation process and sustainability differ based on their managerial level	“Red Apple“ Healthy Food Environments in Hospitals Project (2008, voluntary)	Hospitals (n = 9)	Total (n = 53); senior managers (n = 13), middle managers and dietitians (n = 29), cafeteria supervisors and staff member (n = 11)	Qualitative In-depth face-to-face interviews (sampling only from hospitals that fully implemented the program), field notes	Grounded theory approach (emergent, exploratory, inductive method, elaborate coding process)
San Antonio Sodium Reduction Initiative Sosa et al (2019)^61^; Ullevig et al (2019)^62^ Bexar County, TX, USA	To identify factors affecting implementation of the Sodium-Reduction Initiative	San Antonio Sodium–Reduction Initiative (2013–2016, voluntary)	Workplaces (including hospitals) (n = 8); additionally congregate meal programs (n = 3)	Total (n = 32); food-service operators, wellness coordinators, upper-level management	Mixed methods; longitudinal Semi-structured face-to-face interviews	Thematic analysis
Washington State’s Healthy Nutrition Guidelines Executive Order Otten et al (2014)^57^; Otten et al (2015)^58^; Podrabsky et al (2016)^59^; Podrabsky et al (2018)^60^ Washington state, USA	To develop and conduct an evaluation of the Healthy Nutrition Guidelines To document the experiences of agencies, stakeholders, and venues affected (and, when possible, unaffected) by EO 13‐06 to inform future and continuing implementation and evaluation efforts To capture progress since implementation and provide summative description and overview of progress towards guidelines implementation	Washington State’s Healthy Nutrition Guidelines EO 13‐06 for state facilities (2013, mandatory)	State agencies’ facilities; 2014 (n = 9), 2015 (n = 10), 2016 (n = 23), 2018 (n = 10)	2014: Total (n = 17); agency leaders (n = 9), cafeteria operators (n = 5), worksite wellness coordinators (n = 2), support service representative (n = 1) 2015: Total (n = 31); agency leaders (n = 13), worksite wellness coordinators (n = 11), cafeteria operators (n = 6), vending company representative (n = 1) 2016: Total (n = 23), facility managers and directors as institutional representatives 2018: Total (n = 10); cafe managers (n = 7), vendors (n = 3)	Mixed-methods^c^^,^^d^^,^^e^ yearly evaluation (2017 publication not included) Face-to-face and telephone interviews (2014, 2015, 2018), online implementation survey for state institutions (2016)	Codebooks based on interview responses (aiming for high inter-rater agreement), categorization of responses by topic area
City of Hamilton Policy Atkey et al (2018)^63^ City of Hamilton, Ontario, Canada	To explore the City of Hamilton’s story of municipal Healthy Food and Beverage policy development, as well as key lessons learned throughout this process	City of Hamilton Healthy Food and Beverage Policy (2011)	Municipal buildings and corporate events (number of sites not reported)	Total (n = 2); public health nutritionist (n = 1), healthy workplace specialist (n = 1)	Qualitative Interview (supplemented by further input from authors)	Story development process in health promotion
Healthy Foods in Champlain Hospitals Dojeiji et al (2017)^64^ Champlain region of Eastern Ontario, Canada	To describe implementation strategies and successes, as well as challenges and limitations	Healthy Foods in Champlain Hospitals (2014, voluntary)	Hospitals (n = 20)	Total (not reported); hospital leaders, food service staff, other stakeholders	Not reported Not reported	Not reported
Three Canadian provinces study Kirk et al (2021)^65^ BC, Alberta, and Nova Scotia; Canada	To describe facilitators and barriers to implementing provincial nutrition guidelines in recreation and sports facilities	Nutrition Guidelines for Vending Machines in BC Public Buildings (2007, voluntary, BC); Nutrition Guidelines for the Recreation Sector (2008, voluntary, Alberta); Healthy eating guidelines for recreation and sports settings (2015, voluntary, Nova Scotia)	Recreation and sports facilities (number of sites not reported) across 3 provinces	Total (n = 32); recreation staff managers, facility committee or board members, recreation volunteers	Qualitative Semi-structured telephone interviews; purposive sampling	“Inside out” modified socio-ecological model framework; inductive thematic analysis using qualitative description
Canadian healthy eating in recreation and sport settings McIsaac et al (2018)^66^ Nova Scotia, Canada	To explore stakeholder perspectives on barriers to healthy food provision in recreation and sport settings	Healthy Eating in Recreation and Sport Setting Guidelines (2015, voluntary) or similar	Recreation and sport settings (number of sites not reported)	Total (n = 10); facility general manager (n = 4), municipal recreation manager (n = 5), food and beverage manager (n = 1)	Qualitative Semi-structured telephone interviews; purposive sampling	Qualitative description; inductive open-coding, iterative approach
Healthy Food and Beverage Sales phase I pilot study Naylor et al (2010)^68^ BC, Canada	To evaluate the Healthy Food and Beverage Sales initiative in 10 pilot communities over 6 mo To explore the implementation process and identify common experiences in introducing healthier choices	Healthier Choices in Vending Machines in BC Public Buildings (2007, voluntary); Healthy Food and Beverage Sales Initiative (8 mo) providing training, resources, and technical support	Recreation facilities (n = 41) across 10 pilot communities	Total (not reported); lead project staff (facility staff and contractors) in each community	Mixed-methods,^c^^,^^d^ concurrent triangulation design Documents' review (community applications and final reports), semi-structured telephone interviews	Thematic analysis, iterative process, open coding followed by axial coding and selective coding (interviews); categorization and comparison of goals (document review)
Healthy Food and Beverage Sales phase II study Vander Wekken and Naylor (2010)^70^ BC, Canada	To identify key issues related to implementation of the Healthy Food and Beverage Sales Initiative (phase II)	Healthier Choices in Vending Machines in BC Public Buildings (2007, voluntary); Healthy Food and Beverage Sales in Recreation Facilities and Local Government Buildings Initiative providing training, resources and technical support	Recreation facilities across 17 communities (number of sites not reported)	Total (n = 22); project staff from each community	Mixed methods^c^^,^^d^ Semi-structured telephone interviews	Exploration of common themes
Healthy Food and Beverage Sales controlled study Naylor et al (2015)^67^ BC, Canada	To evaluate the impact of a capacity-building intervention (Healthy Food and Beverage Sales) on organizational capacity for providing healthy food environments, vending products offered for sale and food policy development	Healthier Choices in Vending Machines in BC Public Buildings (2007, voluntary); Healthy Food and Beverage Sales Initiative (8 mo) providing training, resources, and technical support	Recreation and sport facilities (n = 71) in 21 communities receiving intervention and 23 comparison communities	Total (not reported); key recreation and sport facilities staff	Mixed-methods^d^ quasi-experimental, controlled, pre–post comparison design In-depth examination of project proposals, final reports; in-depth semi-structured telephone interviews	Open coding followed by axial coding and constant comparison
Healthy Food and Beverage Sales –Industry Perspectives Vander Wekken et al (2012)^71^ British Columbia, Canada	To explore industry perspectives on the transition to healthier food and beverage sales in publicly funded recreation facilities; specifically the awareness of the BC provincial (state level) guidelines and implementation supports, and challenges encountered in the transition to healthier products	Healthier Choices in Vending Machines in BC Public Buildings (2007, voluntary); Healthy Food and Beverage Sales in Recreation Facilities and Local Government Buildings Initiative providing training, resources and technical support	Recreation facilities (number of sites not reported)	Total (n = 16); representatives of different sectors of the snack and beverage industry: manufacturers (n = 7), suppliers (n = 3), distributors and vendors (n = 5), advocacy and education organization representing nonalcoholic beverage manufacturers (n = 1)	Qualitative Semi-structured telephone interviews	Thematic analysis using framework approach, reciprocal coding followed by axial coding
Healthier Recreation Concession Pilot Project Neil and Haile (2016)^69^ Oxford County, Ontario, Canada	To provide an opportunity to introduce, promote, and evaluate healthy food and beverage sales and uptake within a recreational facility To understand the processes involved with implementing the new menu	Healthier Recreation Concession Pilot Project (6 mo)	Recreational facility (n = 1)	Total (n = 1); concession operator	Mixed-methods^c^^,^^e^ evaluation Telephone interview (open-ended questions)	Content analysis (notes from interview)
South Korean sodium reduction pilot project Lee and Park (2016)^72^ South Korea	To assess the relationships between sodium-reduction practices, barriers, and perceptions among food service personnel	Sodium-reduction pilot project (5 mo)	Type not reported,^f^ total worksites (n = 17)	Total (n = 104); kitchen assistants (n = 63), cook (n = 36), other canteen staff (n = 5)	Quantitative, cross-sectional Self-reported questionnaire (closed questions)	Statistical analyses
South Korean reduced-sodium meals national program Park and Lee (2016)^73^ South Korea	To examine barriers to and facilitators of serving reduced-sodium meals in worksite cafeterias To identify potential policies or programs that need to be developed for lowering sodium content in meals served in worksite cafeterias	Reduced-sodium meals national program (voluntary)	Food companies (n = 10) catering to government-owned facilities and hospitals (and some private companies)	Total (n = 25); on-site dietitians (n = 16), headquarter managers (n = 9)	Qualitative In-depth, face-to-face individual and group interviews	Socioeconomic model framework and preselected constructs used to develop a codebook, a guideline, and a manual for coding
Dutch environmental nutrition program intervention Steenhuis et al (2004)^74^ Netherlands	To identify explanations for the ineffectiveness of the environmental and educational nutrition programs To formulate recommendations for future programs	Environmental nutrition program intervention (6 mo, focus on low-fat products and fruit and vegetables), based on the guidelines for healthy nutrition of the Netherlands Bureau for Food and Nutrition	Government organizations (mainly white-collar workers) (n = 13); included private supermarkets (n = 9)	Total (n = 22); worksite cafeteria managers (n = 13), supermarket managers (n = 9)	Qualitative evaluation of randomized pre-test post-test experimental control group design Semi-structured interviews	Coding and categorization based on interview topics (“cut and paste” technique)
Dutch portion-size and pricing intervention Vermeer et al (2012)^75^ Netherlands	To describe the process evaluation of 2 worksite cafeteria interventions aimed at portion sizes	Portion size with proportional pricing intervention (3 mo)	Hospitals (n = 15), universities (n = 3), police departments (n = 2); additionally included private companies (n = 5)	Total (n = 25) cafeteria managers	Mixed-methods^c^ process evaluation Structured observations (implementation and resources), semi-structured face-to-face interviews (2 mo after intervention ended), telephone interviews (12 mo after intervention)	Diffusion of Innovation theory; semi-structured interviews analyzed using integrated approach (inductive reasoning, and using predetermined code types) presented thematically; remaining data analyzed using descriptive statistics
Scottish Healthcare Retail Standard Stead et al (2020)^76^; Shipton (2019)^77^ Scotland	To examine retailers’ experiences of implementing Healthcare Retail Standard and the impact of implementation on food and drink product range and promotions	Scottish Healthcare Retail Standard (2015, mandatory, national)	NHS hospitals (n = 13) (retail outlets and mobile carts)	Total (n = 32); local shop managers (n = 24), supervisors (n = 4), regional managers (n = 2), business proprietor (n = 1), assistant (n = 1)	Mixed methods^c^^,^^d^ Semi-structured face-to-face interviews (individual and in pairs)	Thematic coding using framework approach based on themes from interview guide and arising from data; triangulation of interview and observational data
Welsh Hospital Vending Welsh Assembly Government (2009)^78^ Wales	To ascertain feedback from individual hospital sites about the implementation of the vending guidance	Health Promoting Hospital Vending Directions and Guidance (2008, mandatory)	NHS hospital sites across 7 health boards (number of sites not reported)	Total (not reported); catering managers, nutritionists	Mixed methods^e^ Questionnaire (optional follow-up visits or telephone interviews to obtain further feedback)	Not reported
East London Food for Life initiative Gray et al (2017)^79^ East London, West Yorkshire, and South Warwickshire, England	To evaluate the impact and challenges of implementing a Food for Life approach within 3 pilot NHS sites in 2014/2015	Food for Life initiative led by the Soil Association (extended to hospital settings in 2013); points-based award system for meeting nutrition standards	NHS Trusts sites (hospitals) (n = 3)	Total (n = 27); strategic managers, hotel services managers, matrons, sustainability leads, staff well-being coordinators, external catering contractors	Qualitative case study Semi-structured interviews (30–60 min), documents' analysis (action plans, meeting notes, communication materials)	Thematic analysis informed by theory of change approach
“Healthful & Tasty: Sure” Sodium Reduction Trial Beer-Borst et al (2018)^80^ (protocol); Beer-Borst et al (2019)^81^; Beer-Borst et al (2020)^82^ Switzerland	To demonstrate the effectiveness of a combined educational and environmental intervention in the workplace in reducing employees’ average daily sodium/salt intake To enhance guideline compliance among the catering facility team To examine the extent to which communal catering team coaching was able to trigger action and offer potential for a supporting nutritional environments	“Healthful & Tasty: Sure!” (Gesund & Gut: Na Klar!) Health Promotion Sodium Reduction Trial (May 2015–November 2016); applying recognized national guidelines for communal catering	Manufacturing and service industry (n = 2), administration and offices (n = 2), university and research institutions (n = 2), social service and welfare institution (n = 2); both private and public organizations	Total (n = 22); individual roles not reported	Mixed-methods^c^ cluster, nonrandomized, single-arm trial with calibration arm Catering surveys, online questionnaires	Descriptive statistics
Danish “6-a-day” Worksite Canteen Study Lassen et al (2004)^83^ Denmark	To gain knowledge of practical strategies being effective in increasing the consumption of fruits and vegetables in worksite canteens	Danish “6-a-day” Worksite Canteen Model Study (launched 1999)	Military base (n = 1), town hall (n = 1), waste-handling facility (n = 1), private workplaces (n = 2)	Total (not reported), canteen managers	Mixed-methods model study Self-reported sharing of ideas and lessons learned	Not reported
New Zealand Heartbeat Programme Young et al (2004)^84^ New Zealand	To determine the effect of the Heartbeat Catering Program on the provision of healthy menu items by measuring perceptions of caterers and dietitians involved in the program To review program resources and services in order to plan future program directions	Heartbeat Catering Program and Guidelines (1992, voluntary), Function Catering Guidelines (1994, voluntary), Recipe Development Guidelines (1999, voluntary)	Workplace cafeterias that used catering (included private companies) (number of sites not reported)	Total (n = 179); caterers and food service managers (n = 164), dietitians (n = 15)	Mixed methods Postal questionnaire, telephone descriptive interviews, in-depth structured interviews (telephone and in-person)	Not reported

a Includes information on policy type, approach, and year if reported in the studies.

b As reported by the study’s authors.

c Includes customer surveys.

d Includes results of food environment audits by study team or self-reported by study settings.

e Includes analysis of sales data.

f Unsuccessful attempt at contacting study author for clarification.

*Abbreviations:* ACCHO, Aboriginal Community Controlled Health Organisations; BC, British Columbia; DFAC, dining facility; EO, Executive Order; NHS, National Health Service; WA, Western Australia.

Most of the studies (n = 35) assessed barriers and facilitators to implementation of formal and informal policies, guidelines, standards, and initiatives adopted or followed by organizations where the study took place. In the case of some studies, it was unclear whether the policy guidelines were voluntary or mandatory. Specific details of the policies (where description was included in the published records) varied in the nutritional values or cut points used for determining whether foods or drinks were healthy or unhealthy, and in the proportions of healthy vs unhealthy foods that should be available in the food outlets and vending machines or offered during catering. The remaining 6 studies conducted worksite interventions aimed at sodium reduction aligning with national guidelines (n = 3),^72^^,^^73^^,^^80–82^ increasing fruit and vegetable offerings (n = 1),^83^ combined with a focus on low-fat products (n = 1),^74^ and adjusting portion sizes (n = 1).^75^

Data on barriers and facilitators were collected from 1307 participants across 32 studies that reported their sample sizes, with numbers ranging from 1^69^ to 179 participants^84^ per study. Although some studies (n = 9) did not report participant numbers, most studies (n = 39) provided at least a basic participant description, often representing a mixture of role types. Studies were conducted with facility managers, senior managers, executives, and participants in similar roles (n = 20); food service owners, retailers, and food service or canteen managers (n = 19); nutritionists and dietitians (n = 11); worksite staff in charge of well-being, wellness, or health promotion (n = 8); cooks, head chefs, kitchen assistants, and other food service staff (n = 7); caterers and catering staff (n = 5); external contractors, suppliers, and manufacturers (n = 4); and procurement managers (n = 2).

Mixed-methods studies were predominant in this review (n = 25). Qualitative design was solely used in 12 studies, and solely quantitative design in 2 studies. Two studies did not explicitly report on the study design.^51^^,^^64^ Individual, pair, and group interviews were the most commonly used methods to gather data relevant to this review in 30 studies and were conducted either over the telephone or in person (n = 19), but this information was not further specified in 11 studies. Other methods used were surveys and questionnaires (n = 12), analysis of relevant documents (n = 6), focus groups (n = 1), structured observations (n = 1), workshops (n = 1), self-report implementation templates (n = 1), and self-reporting and sharing of ideas and lessons learned (n = 1). Two studies did not report the methods used to collect the data.^51^^,^^64^ Large variation was seen with regard to methods used to analyze data on barriers and facilitators to policy implementation. However, 9 studies did not specify the methods used to analyze the data.^39^^,^^47^^,^^50–52^^,^^64^^,^^78^^,^^83^^,^^84^ Generally, there was a lack of detailed description of the analytic methods used or steps taken to analyze the data.

## RESULTS OF THEMATIC SYNTHESIS OF FINDINGS

Four themes were generated pertaining to barriers and facilitators to implementation of and compliance with healthy food and drink policies aimed at the general adult population in public sector workplaces (summarized in Table 3). Studies reported multiple barriers and multiple facilitators to implementation, with participants’ perspectives reflecting their level of involvement. The themes generated represent patterns of shared meaning across all of the included studies in this review.

**Table 3:** Themes and subthemes identified in the review

Theme 1: A ratified policy as the foundation of a successful implementation plan
Nature of healthy food and drink policy
Successful implementation and maintenance plan
Availability of internal and external support
Theme 2: Food providers’ acceptance of implementation is rooted in positive stakeholder relationships, recognizing opportunities, and taking ownership
Building rapport with food providers
Managing customer expectations
Recognizing opportunities and taking ownership of implementation
Theme 3: Creating customer demand for healthier options may relieve tension between policy objectives and business goals
Alignment between policy objectives and food providers’ goals
Facilitating customer demand for healthier options
Theme 4: Food supply may limit the ability of food providers to implement the policy
Food supply-related factors influencing policy implementation
The ease of change from unhealthy to healthy offerings

### Theme 1: A ratified policy as the foundation of a successful implementation plan

#### Subtheme: Nature of healthy food and drink policy

The first theme focuses on 3 important elements of an effective workplace healthy food and drink policy implementation framework. First, a clear, consistent, and evidence-based policy that is mandatory or endorsed by local or national government and senior workplace management is the foundational element (or first step) in successful implementation. As a participant in Western Australia noted, “it helps when things come from the top down. It provides momentum and makes it a priority.”^47^ Formal recognition of healthy food and drink policy is also often ratified by including a policy compliance clause in all retailer and food service contracts with “compliance being a condition of the…contract with the retail business.”^76^ Official endorsement signals the importance of the policy to all stakeholders and gives authority and autonomy to implement the policy and enforce, evaluate, and monitor compliance. Additionally, it safeguards continuation of implementation and compliance in case of retailer or food service operator changes.

On the other hand, a voluntary or unofficial healthy food and drink policy, or a lengthy endorsement process, represents little commitment from government or senior workplace management and may hinder or impede successful implementation. As MacDonald et al stated, “the lack of formal endorsement of the policies by the management…casts some doubt over the strength of their commitment.”^42^ A lack of a healthy food and drink policy compliance clause in food provider contractual agreements does not provide a strong enough (often legal) basis to enforce policy implementation and compliance. Furthermore, “a change in vendors may nullify any progress made if the new vendor decides against implementing [voluntary guidelines],”^39^ and additional policy-related barriers to implementation can also derive from ambiguous or open to interpretation policy components or criteria.

#### Subtheme: Successful implementation and maintenance plan

The foundational document must be accompanied by the second element, a comprehensive, realistic, and goal-driven implementation and maintenance plan tailored to individual workplaces and guided by policy objectives and criteria. Communities in British Columbia believed strategic planning “would provide a solid foundation for a long-term commitment to providing healthy food environments within their recreation and sports facilities.”^67^ Baseline assessment, piloting, and/or learning from other settings allow the development of an enabling strategy with a simple, feasible, and gradual change approach that exerts pressure on and supports food providers to achieve policy compliance. Goals, priorities, and time frames will differ between workplaces, even when the same healthy food and drink policy is adopted or mandated. Participants in the study by Blake et al “expressed that an incremental or stepwise change approach had been crucial to effectiveness…[and]…emphasised the importance of learning from experience through trial and error and adaptation.”^32^ Thus, a successful implementation plan accounts for and is adaptive to readiness to change, availability of resources, and possible opportunities and challenges. Regular, planned evaluations of implementation and compliance provide information on achievements, unpredictable challenges, unintended consequences, and resource requirements. As Atkey et al stated, “for concrete change, consistent monitoring of the policy and efforts to keep the policy at the forefront of the [organization’s] everyday business practices are necessary.”^63^ They also can be used to adapt implementation strategies and guide pathways to full compliance.

However, not having an implementation plan or having a plan that is overly complex, unrealistic, or dependent on unavailable resources increases the risk of failure to achieve the desired changes. Without a tailored and feasible plan, it may be difficult to recognize challenges as they arise or implementation efforts that are too small to influence change or have reached saturation point. During implementation “changes do not happen in isolation—it takes time and there are ripple effects from 1 challenge to another,”^70^ which may result in compounding implementation challenges. Furthermore, impractical, one-off, or sporadic implementation or evaluation activities are unlikely to provide useful information on successes and challenges, and limited justification for policy revisions, implementation plan adaptations, and dedication of new or continued supportive resources.

#### Subtheme: Availability of internal and external support

The third important element in successful implementation is support, both internal and external support available throughout the implementation period, regardless of whether the policy is mandatory or voluntary. The level and extent of an individual organization’s support needs can be identified via piloting the policy, formulating an implementation plan, and conducting regular evaluations. Studies often noted that a committed and supportive internal leadership team openly endorsing and following the policy themselves was crucial for successful implementation. Lassen et al stated that “first of all, the attitude of management is central to overcome barriers to change.”^83^ Leadership support can be demonstrated through various actions, such as active involvement in implementation activities, assistance in tackling challenges, allocation of financial resources, dedication of staff time, and appointment of an implementation champion or team with clear roles and responsibilities. There may be a need to “reinforce to management their role in ensuring strategy implementation. This needs to come from a higher authority,”^44^ such as local or national government-legislated healthy food and drink policies or directives.

An important part of such support is ongoing tailored assistance from nutritionists or dietitians to guide food providers through the policy criteria and implementation actions, such as classification of foods and drinks, nutritional analysis, or recipe adaptations. Food providers in the repeated evaluation study in Washington state “appreciate and desire technical assistance and support in both understanding the [guidelines], and in identifying changes they can make and products they can stock in order to be in compliance.”^60^ The degree to which a public organization can effectively support implementation may vary, although many workplaces may rely on external support provided by a state or national government, or existing academic or nutrition-focused organizations. In a US study, “having both internal and external partners contributed to successful healthy vending policies and initiatives. Two cities [of 4] described previous efforts to implement a vending policy that was unsuccessful as a result of not having support from other government departments and community partners.”^52^ External support can have a better reach and “facilitate scale-up of these initiatives”^33^ through access to nutrition professionals not otherwise available in a workplace, and development and provision of centralized, readily available, policy-specific tools and resources.

Additional paper-based, digital, or online support aids may facilitate implementation. These include implementation toolkits or manuals, self-evaluation tools, scalable sample recipe ideas, training to improve staff culinary skills, newsletter and contract templates, access to nutrition analysis software, or a database listing healthy, compliant products. For example, participants in the Law et al study identified a “centralised provision of lists or databases of classified foods and drinks to reduce duplication of effort and the technical burden,”^41^ because food providers noted, “You can’t go and find out this stuff yourself so easily.”^70^ Importantly, such tools and resources need to be tested, user friendly, and up to date.

Little support or engagement from leadership or government, lack of or limited staff resources, high staff turnover, competing demands and responsibilities in the workplace, and time constraints were commonly cited barriers, often combined with a lack of financial resources. Implementers supporting food providers that were not in managerial positions and those from under-staffed departments often had little impact on changes and lacked motivation and energy to become involved in implementation. “The bare-bones nutrition workforce at each site, their limited policy influence and time to engage in the project”^42^ were significant challenges in the Aboriginal Community-Controlled Health Organizations policy implementation. Therefore, implementation may be challenging “without resources and support from key internal and external system players.”^56^ Additionally, implementation barriers can stem from a lack of, outdated, or low awareness of existing tools and resources among support staff and food providers, or from tools and resources that are impractical and nonessential.

### Theme 2: Food providers’ acceptance of implementation is rooted in positive stakeholder relationships, recognizing opportunities, and taking ownership

#### Subtheme: Building rapport with food providers

The second theme centers on different but interdependent pathways to creating acceptance of implementation among food and drink providers who may instinctively resist any external attempts at restricting their usual offerings. Although a positively framed policy and implementation plan are advantageous in lessening negative perceptions of change, accepting change requires building rapport with food providers, contingent on existing and new positive relationships in a workplace. As Law et al noted, “the trust built with the PHD [public health dietitian] meant [food providers] openly discussed their experiences, from a position of acceptance, not resistance.”^41^ Studies frequently identified open and clear communication between stakeholders as facilitating the shift from food providers’ resistance to acceptance, for example, by “making sure that everybody knew all along the way what we were doing, what was expected of them and of their team.”^53^ Regular communication efforts originating from governmental bodies, senior workplace management, or project champions demonstrate acceptance across organizational levels, and indicate to food providers the leadership’s willingness to work collaboratively to overcome barriers and achieve policy compliance. Green et al highlighted “that a positive relationship and open, frequent communication where both the city and vendor listened to each other’s concerns and worked together to address them was essential to successful implementation.”^52^ Furthermore, shared responsibility and a collaborative approach to implementation can mitigate feelings of being disproportionately disadvantaged as a food provider and allows recognition of implementers’ fears, concerns, and barriers (both experienced and perceived) that can create negative perceptions of the policy.

However, when food providers, chefs, and cooks see the approach as a prescriptive rather than as a collaborative initiative, the process may create negative attitudes toward implementation and result in difficulties building rapport and getting all food providers on board. Park and Lee suggested “positive incentives for caterers and other companies to provide healthier options…can be more effective than controlling them with strict rules and regulations.”^73^ Nonexistent or sporadic communication efforts may generate feelings of confusion (especially when stakeholders have little nutrition background), and the implementation may be experienced as overwhelming, isolating, and challenging by individual food providers and may lead to stakeholders being unsure of their roles and responsibilities. As Otten et al summarized, “a lack of communication, knowledge, and understanding throughout the system appear to create barriers towards implementation.”^57^ Communication may be more resource intensive in larger organizations with numerous food providers and outlet operators and in cases where food providers are unwilling to cooperate and resist change. This may result in sluggish progress in building positive relationships.

#### Subtheme: Managing customer expectations

Food providers’ personal views and attitudes toward implementation may be underpinned or modulated by relations with and feedback from customers that influence the extent to which customers are the driving or hindering force in implementation. Customers were often perceived not only as not wanting or not being ready for healthier options but also as expecting unhealthy foods and drinks in public settings. These perceptions were often associated with the “cultural context that less healthy foods were a ‘treat,’ despite their ubiquity,…[and]…their provision was attributed to patrons wanting the less-healthy options.”^66^ Customer resistance to change was a barrier to implementation in public sector workplaces, although the major source of customer complaints generally related to limitations on familiar, unhealthy comfort foods and drinks rather than additions of the healthier items. One food provider even described customers’ criticism as “abuse”^41^ toward them or their front-of-house staff. As studies included in the review highlighted, dissatisfaction with removal of or restrictions on unhealthy items may be attributed to personal eating habits and purchasing patterns that are hard to change, particularly “if people have eaten this way their whole lives.”^56^ The perceived customer resistance was taken, at times, as justification by food providers for noncompliance with policy guidelines. In a US hospital, a food provider acknowledged choosing the path of least resistance with his customers, stating “We took out the fries, and we still hear about it to this day and it’s been over 2 years ago. That’s why I didn’t take out the sugar-sweetened beverages, because I didn’t want to hear about that.”^54^ However, there was also customer acceptance of and support for the move to healthier options, manifested by a perceived shift in customer purchases from unhealthy to healthy products and positive feedback on implemented changes. Through surveys, Humphrey Building employees “expressed a desire for healthier and more sustainable food choices and practices,”^49^ and in Australia, a head chef noted “you can see lines at the salad bar at lunchtime, and you can see that the hot [fried] side’s not as busy.”^36^ Positive customer feedback, especially on initial changes to usual food offerings, is likely to increase food providers’ willingness to implement policy criteria.

Nevertheless, customers' perceived, expected, or actual negative reactions may be disheartening to managers and food service staff at all levels and reinforce their negative perceptions of implementation. Additionally, implementation may be perceived as infringing on the freedom to make personal choices about the foods and drinks that can be offered (for food providers) and purchased (for customers), generally coupled with food providers’ views that responsibility to change behaviors or force adult customers to eat healthily should not be placed on their shoulders. Thus, food-outlet operators may see their business’s primary purpose being “to serve what their customers want, not to dictate what is healthy or not for them”^58^ and may perceive their roles as limited in influencing or convincing customers to buy and consume healthier food and drink. However, convincing food providers that transition to healthier foods offers different choices for their customers where there may have previously been no other choices than unhealthy options may be seen as caring and can increase the positive perceptions of their own roles in providing healthy choices and improving customers’ health.

#### Subtheme: Recognizing opportunities and taking ownership of implementation

Food providers’ acceptance or resistance to implementation may stem from and be reinforced by the observed or assumed acceptance or resistance from customers, although it may also echo across food providers and leadership within an organization, creating multilayered enablers or barriers. The shift toward acceptance can be facilitated by creating and highlighting opportunities associated with implementation and by empowering rather than forcing food providers to take ownership of their roles and implementation actions. Becoming a leader in providing healthier foods and drinks, rebranding or creating a new positive image of compliant food outlets, and using preexisting workplace wellness culture are some of the opportunities that may facilitate implementation. Perceiving policy implementation “as [the] catalyst that could provide the vending industry the opportunity to shed its junk food reputation and enter the future with a more positive, healthier image”^71^ suggests such opportunities may also exist across the food supply-chain.

Creating and recognizing opportunities can naturally align with solution- rather than problem-based attitudes. However, this may require implementers to adopt a fresh perspective to identify novel ways of overcoming barriers, such as increased workloads on food service and retail staff. As Armstrong et al reported, through “creating this additional [food preparation] shift, the DFAC [a military dining facility] manager adapted to this [ingredient preparation] challenge demonstrating that this kind of flexibility is essential for successful intervention implementation.”^48^ The challenges involved in implementing change may diminish over time as food providers gradually increase their autonomy while gaining personal satisfaction from successfully implemented changes and may find “that implementation wasn’t as difficult as they had initially thought it would be.”^77^ Local data and statistics on nutrition-related disease rates can be used to generate acceptance among food providers who may recognize healthy eating as important in the local context, especially if their outlets are located in hospitals or sport and recreation centers that naturally align with health promotion messages. Acceptance may also fade over time, but food providers’ belief that they are a part of a bigger movement or change to improve public sector food environments, “and the fact that everyone else is doing it”^67^ can be empowering and facilitate ownership and implementation.

### Theme 3: Creating customer demand for healthier options may relieve tension between policy objectives and business goals

#### Subtheme: Alignment between policy objectives and food providers’ goals

Intrinsic motivators and values of stakeholders involved in or affected by policy implementation can differ and create tension within an organization. For policy makers, governmental bodies, and senior workplace management, policy objectives represent a way to improve food environments in public sector workplaces, leading to a positive health impact on employees and the wider population. However, policy objectives may not align with food providers’ goals to offer food and drink that meet their customers’ demands and generate profits. Being profitable is often the most important consideration for food providers because profits are a key measure of business success and the basis of their incomes. Anticipated or actual lower sales and profits as a consequence of providing policy-compliant items are a source of concern and a significant barrier and may lead to reluctance by food providers to attempt implementation actions due to “fear…that revenue would be lost if they sold healthier foods and/or stopped selling less healthy foods.”^66^ Associated with this tension is perceived or actual loss of customers who could purchase unhealthy food from competing outlets (within or nearby a workplace) that do not need to comply with the policy. In the study by Kirk et al, “some participants expressed that changing the food environment in their facility to include healthier options was risky if there were (or were perceived to be) unhealthy alternatives close by.”^65^ Although a decrease in sales can have an obvious impact on the overall profitability, there may also be extra responsibilities and an increase in costs of running a policy-compliant food outlet, which can be higher for smaller businesses.

Additional labor costs, staff training, and new cooking and storage equipment purchases, combined with the common belief that healthier foods and drinks have lower profit margins, further reiterate the likelihood of profit losses, which also has a ripple effect on workers fearing loss of jobs. Law et al pointed out that “although healthy food procurement policies are considered low-cost to governments, there is a cost of implementation borne by food retailers.”^41^ Pressures to generate profits and run a commercially viable business may also stem from tight budgets, sponsorship arrangements, and revenue targets set out in contracts, contributing to the overall financial barriers. It can be “difficult for healthy food to be as profitable as unhealthy food, and doing so takes more resources and effort.”^32^ Hence, the implementation team should work with food providers to identify ways to minimize operational costs, and leadership should allocate funding for any necessary new equipment or food service staff training. Diversifying revenue streams to include nonfood and nondrink items, moving vending machines to higher-traffic areas, and analyzing sales data to determine the most and least profitable operational hours and popular healthy meals are examples of ways to mitigate profit loss. However, creating customer demand for healthier options is essential in ensuring “financial viability [that] allows for confidence in changes”^39^ and decreasing tension between policy objectives and profitability.

#### Subtheme: Facilitating customer demand for healthier options

Customer acceptance of a healthy food policy does not necessarily translate to a demand for healthier options, because intrinsic motivators during purchasing decisions were often perceived to be primarily taste, cost, and familiarity, not the nutrition value or health consequences of food and drink. In Canada, compliant healthy items “often did not sell because consumers found them unfamiliar, unpalatable or simply too expensive.”^71^ Thus, even though healthy products are available, they might be “much less popular with customers compared to [less healthy] ‘red’ and ‘amber’ foods,”^34^ and health-conscious customers may already bring their food in from elsewhere. Assuming customers will readily accept changes and switch to healthier products, especially when they expect certain kinds of food in the workplace, combined with little attempt to gain their support, can create an implementation barrier. However, rather than expecting customers to choose healthier options, the demand can be facilitated, relieving tension between policy objectives and food providers’ goals. Conducting customer surveys before and during implementation may be helpful to identify actual customer preferences, desirable healthy options, and specific employees’ needs, such as shift or night workers.

Offering small taste-size samples or organizing taste-testing sessions of healthier options are good strategies “to introduce new products and let customers try samples before buying,”^53^ gather feedback, and “win people over, generate support, and overcome negative perceptions.”^63^ Furthermore, having tasty and acceptable food and drink in place before limiting or removing popular unhealthy options, especially in the early stages of implementation, could reassure customers they still can make their own food choices while gradually exposing them to healthier options. Such facilitating approaches combined with active, complementary, and multicomponent health promotion and marketing strategies aimed at employees and visitors can increase customer awareness, support, and, ultimately, demand for healthier options.

Regular informative and promotional messages directed at customers need to be captivating, tailored to workplace and cultural contexts, and communicated consistently across all platforms, such as slogans, posters, color-coded product labels and visual cues, as well as social media posts, newsletters, intranet, and staff emails. As 1 participant noted, “the marketing and education…you cannot underestimate the importance of those pieces”^49^ in creating customer demand, and this was reiterated by studies identifying little “engagement with customers…as a missed opportunity”^77^ and the existence of “often little communication in areas with high staff resistance.”^44^ Additionally, subtle actions focusing on the positive aspects of healthier food help mitigate profit losses, retain current patrons, and attract new, health-oriented customers. Facilitating pricing strategies reported in studies included competitive pricing and discounting on healthier options, offering meal deals or food loyalty programs solely on policy-compliant items, and gradual price increases of unhealthy options. Choice architecture, such as intentional food and drink display arrangements and focused lighting, was used by food providers to normalize policy-compliant items and make them prominent “to bring them [healthy food] to the front of customers mind”^78^ while making less healthy options only available on request. However, a lack of or limited efforts to facilitate customer demand result in persistent challenges to implementation.

### Theme 4: Food supply may limit the ability of food providers to implement the policy

#### Subtheme: Food supply–related factors influencing policy implementation

Successful sourcing of policy-compliant products by food providers, with or without the help of nutritionists or dietitians, facilitates implementation of a healthy food policy. Suppliers willing to collaborate and renegotiate their contracts with food providers to stock healthier items, increase and diversify their healthy product range, and even classify items against policy criteria for quick selection are enablers of change. Additionally, looking for new procurement avenues, especially among small local businesses, can provide a more comprehensive selection of healthy food and drink options while allowing food providers to negotiate and work directly with local producers and suppliers. As 1 food service director stated, “We sat down with our vendors and said we needed them to make a recipe that cuts sodium content in 10 breads in half. They did it. Within 3 weeks we had a new recipe and we had healthier items.”^54^ However, procurement can vary between different organizations and businesses, and the process can be an implementation barrier when it is complex, time consuming, and involves many stakeholders. As Blake et al stated, “identifying and negotiating with new and existing suppliers to provide healthier options was a major time commitment and created logistical challenges.”^32^ Restrictions may be included in procurement contracts that prevent sourcing of products from different suppliers, while minimum product order requirements may leave smaller food providers without enough buying power to purchase healthier, less popular options. Furthermore, existing contractual agreements may include agreed-upon promotional and marketing arrangements and incentives, combined with pressure from suppliers’ representatives to purchase unhealthy products.

Thus, the food supply chain has a substantial role in implementation. Some of the significant supply chain–related barriers include limited availability and range of healthier products on the market, making them hard to find, and a lack of distribution chain from manufacturers to food providers or inconsistent supply of healthy products already available. According to Steenhuis et al, “Cafeterias which wanted to increase their range of low-fat products had difficulty in obtaining them. Their regular suppliers and wholesalers could not always supply the new products, or provide them on a daily basis.”^74^ The characteristics of healthier items may also create implementation challenges, especially shorter shelf-life of healthier products, because manufacturers, suppliers, or food providers may perceive these items as too perishable, leading to high food wastage. “It’s not so difficult if it is a frozen item, because shelf life is longer, but if it is a fresh item…[the] distributor is very reluctant to bring this product in,” noted Bayne et al.^49^ Additionally, size restrictions set out in a policy can make healthier products with large single-unit sizes noncompliant, while easily breakable or nonstandard product packaging sizes may not be suitable for specific food settings such as vending machines.

Just as retailers are restricted by supply-side availability, suppliers are limited by manufacturing trends. Development of new products or healthier reformulation of current products are time and resource intensive and have been identified as costly by manufacturers “and in many cases threatened business survival.”^71^ However, increased buying power in the supply chain and an increase in market demand seem to incentivize manufacturers to produce healthier products. Adoption of healthy food and drink policies across multiple settings, such as public sector workplaces, government agencies, or education facilities, creates markets for food-supply chain stakeholders, often with untapped business potential, and may lead to expansion of policy-compliant product ranges. For example, in Canada, “suppliers and distributors had begun to provide a ‘healthy products’ selection and to reconfigure size and content to comply with earlier released school guidelines. This made it easier for the recreation facilities to work with vendors and make changes.”^68^ With the food industry also basing its actions on market demands, perceived public shift to be more health conscious, with an increasing focus on healthy eating, is a motivating trend and driver for manufacturers to provide healthier products. Additionally, voluntary or mandatory healthier reformulation targets set out by government bodies are likely to exert pressure on manufacturers and thus facilitate overall changes in the national food supply, especially if supported by governmental grants. Still, manufacturer-related barriers to implementation persist, reflected in difficulty by food providers to find policy-compliant products that may not yet exist on the market, as well as discontinuation of currently suitable products.

#### Subtheme: The ease of change from unhealthy to healthy offerings

Location, size, layout, and type of workplace and food outlets may be barriers to as well as facilitators of policy implementation. Workplaces located in metropolitan and higher socioeconomic areas have access to more suppliers and delivery routes but may also have several unhealthy food outlets situated nearby. “Agencies with locations in more rural areas do not have the same food resources available as those in urban areas where there is more competition among food suppliers.”^58^ Based on available resources, buying power, and high customer numbers ensuring stock turnover, larger workplaces and food outlets may find implementation less challenging, although smaller size facilities often have less complicated structures and processes that may enable more rapid changes toward compliance. However, limited space and impractical physical configurations and layouts are implementation barriers regardless of location and size. In Scotland, “most shops had to reconfigure their layouts and planograms to adapt to the [guidelines], in some cases also having to make physical changes to shelving units, chillers and other fixtures.”^76^ Difficulties removing deep fryers, costly changes to rearrange and modify refrigeration and cooking equipment, and an inability to move wall and floor fixtures were often cited as challenges.

Some food outlets may face particular challenges to implementation of healthy food policies. Although settings with mostly unpackaged foods that prepare foods on site, such as staff cafeterias and cafes, seem to have higher labor and training costs associated with implementation, they can readily modify their recipes to provide healthier versions of the foods and drinks currently on offer that their customers find familiar. However, “if there are no pre-tested…recipes that chefs can use immediately, it is difficult for onsite staff to develop these recipes.”^73^ Standardized recipes ensure consistency in meeting policy criteria, with compliance often achieved by cooking from scratch using healthy but less costly ingredients, switching to healthier preparation and cooking methods, and increasing vegetable, fruit, and herb content of meals. On the contrary, outlets with mostly or only packaged foods, such as kiosks and vending machines, that outsource all their foods may find it challenging to comply with guidelines. Although healthier drink options are likely familiar and popular, resulting in higher healthy-beverage implementation rates, finding healthier packaged snacks and meals seems more challenging, especially for vending machines. In Western Australian evaluation, “‘drinks only’ vending machines were most likely to meet all Policy requirements (83.7%) and ‘food only’ vending machines were least compliant (6.3%).”^47^ Current design of vending machines can inhibit stocking of policy-compliant items, and slot modifications to fit new healthier products can be slow and costly because they need to be undertaken by a trained professional. As a result, complete removal of food- and drink-dispensing machines was often a reported unintended consequence of healthy food and drink policies.

### Methodological strengths and limitations of included studies

The methodological limitations of studies ranged from negligible to major (Table 4).^32–84^ Studies published in peer-reviewed and scientific journals did not necessarily show more robust methodology when compared with grey literature studies and reports. There were 20 studies with negligible or minor limitations, and 21 studies with moderate or major limitations. Noted strengths (in most studies) were an ethical approval statement, use of appropriate study design, clear presentation of findings, and considerations of the study’s contribution to the research field. However, a lack of or inadequate description of data collection and analysis methods, and scarcity of information on the relationship between researcher(s) and participants were the main areas of methodological limitations. Specifically, reports on the qualitative data analysis processes were frequently brief, stating only general terms for a chosen method, such as thematic analysis or content analysis, or software used, with no details or further explanations of the steps undertaken by researcher(s) to analyze data.

**Table 4:** Quality assessment of methods related to barriers and facilitators of implementation in the included studies

Reference	Clear aims	Methodology appropriate	Research design appropriate	Recruitment strategy appropriate	Data collection appropriate	Relationship adequately considered	Ethical issues considered	Data analysis rigorous	Clear findings	Research valuable	Methodological limitations
Healthy Choices in Victorian Recreation Centres Blake et al (2021)^32^	Y	Y	Y	Y	Y	U	Y	Y	Y	Y	Minor
Healthy Choices in Alfred Health (2017) Boelsen-Robinson et al (2017)^34^; Peeters et al (2017)^37^; Boelsen-Robinson (2019)^33^	Y	Y	Y	Y	Y	U	Y	Y	Y	Y	Minor
Healthy Choices in Alfred Health (2019) Boelsen-Robinson et al (2019)^36^; Victorian Health Promotion Foundation (2017)^38^; Boelsen-Robinson et al (2016)^35^; Boelsen-Robinson (2019)^33^	Y	Y	Y	Y	Y	U	Y	Y	Y	Y	Minor
Victorian Healthy Choices and Healthy Catering case studies Chang et al (2016)^39^	Y	Y	U	U	U	U	U	No	Y	Y	Major
Healthy Choices in Aboriginal ACCHOs MacDonald et al (2016)^42^	Y	Y	Y	Y	Y	U	Y	U	Y	Y	Moderate
Victorian policies in sport and recreation facilities Riesenberg et al (2020)^45^	Y	Y	Y	Y	Y	U	Y	Y	Y	Y	Minor
A Better Choice evaluation Miller et al (2015)^43^; Queensland Health (2010)^44^	Y	Y	Y	Y	Y	U	Y	U	Y	Y	Moderate
Queensland children’s hospital study Walker et al (2020)^46^	Y	Y	Y	Y	Y	Y	Y	U	Y	Y	Minor
Healthy Options WA Department of Health, evaluation Western Australia Department of Health (2020)^47^	Y	Y	Y	U	U	U	U	No	Y	Y	Major
Healthy Options WA in East Metropolitan Health Service Law et al (2021)^41^	Y	Y	Y	Y	Y	Y	Y	Y	Y	Y	Negligible
South Australian Health facilities evaluation Government of South Australia (2012)^40^	Y	U	U	Y	U	U	U	No	Y	U	Major
Army DFACs intervention Armstrong et al (2021)^48^	Y	Y	Y	Y	Y	Y	Y	Y	Y	Y	Negligible
Hubert Building Cafeteria Guidelines Bayne et al (2012)^49^	Y	Y	Y	Y	Y	Y	Y	U	Y	Y	Minor
Boston Sodium Reduction Initiative Brooks et al (2017)^50^	Y	No	No	No	No	U	Y	No	No	Y	Major
Vidant Healthy Food Environment Policy Gaskins et al (2013)^51^	Y	No	No	No	No	U	U	No	No	Y	Major
Healthy Vending Policies in US cities Green et al (2020)^52^	Y	Y	Y	U	U	U	Y	U	Y	Y	Major
US Hospital and Federal Worksites Guidelines Jilcott Pitts et al (2016)^53^	Y	Y	Y	Y	Y	Y	Y	Y	Y	Y	Negligible
US hospital food service guidelines Jilcott Pitts et al (2018)^54^	Y	Y	Y	Y	Y	Y	Y	Y	Y	Y	Negligible
New York City Healthy Hospital Food Initiative Lederer et al (2014)^55^	Y	Y	Y	Y	Y	U	Y	U	Y	Y	Moderate
Red Apple Hospitals Project Neffa (2011)^56^	Y	Y	Y	Y	Y	Y	Y	Y	Y	Y	Negligible
San Antonio Sodium Reduction Initiative Sosa et al (2019)^61^; Ullevig et al (2019)^62^	Y	Y	Y	Y	Y	U	U	U	Y	Y	Major
Washington State’s Healthy Nutrition Guidelines Executive Order Otten et al (2014)^57^; Otten et al (2015)^58^; Podrabsky et al (2016)^59^; Podrabsky et al (2018)^60^	Y	Y	Y	Y	Y	U	Y	Y U (2018 report)	Y	Y	Minor
City of Hamilton Policy Atkey et al (2018)^63^	Y	Y	Y	Y	U	Y	Y	U	Y	Y	Moderate
Healthy Foods in Champlain Hospitals Dojeiji et al (2017)^64^	Y	U	U	U	U	U	U	U	Y	Y	Major
Three Canadian provinces study Kirk et al (2021)^65^	Y	Y	Y	Y	Y	U	Y	Y	Y	Y	Minor
Canadian Healthy Eating in Recreation and Sport Setting McIsaac et al (2018)^66^	Y	Y	Y	Y	U	U	Y	Y	Y	Y	Moderate
Healthy Food and Beverage Sales phase I pilot study Naylor et al (2010)^68^	Y	Y	Y	Y	Y	U	Y	Y	Y	Y	Minor
Healthy Food and Beverage Sales phase II study Vander Wekken and Naylor (2010)^70^	Y	Y	Y	Y	Y	Y	U	U	Y	Y	Moderate
Healthy Food and Beverage Sales study Naylor et al (2015)^67^	Y	Y	Y	Y	Y	U	Y	No	Y	Y	Moderate
Healthy Food and Beverage Sales -Industry Perspectives Vander Wekken et al (2012)^71^	Y	Y	Y	Y	Y	U	Y	Y	Y	Y	Minor
Healthier Recreation Concession Pilot Project Neil and Haile (2016)^69^	Y	Y	Y	Y	Y	U	U	U	Y	Y	Major
South Korean Sodium reduction pilot project Lee and Park (2016)^72^	Y	Y	Y	Y	Y	U	Y	Y	Y	Y	Minor
South Korean reduced-sodium meals national program Park and Lee (2016)^73^	Y	Y	Y	Y	Y	U	Y	Y	Y	Y	Minor
Dutch environmental nutrition program intervention Steenhuis et al (2004)^74^	Y	Y	Y	Y	Y	U	U	Y	Y	Y	Moderate
Dutch portion size and pricing intervention Vermeer et al (2012)^75^	Y	Y	Y	Y	Y	U	Y	Y	Y	Y	Minor
Scottish Healthcare Retail Standard Stead et al (2020)^76^; Shipton (2019)^77^	Y	Y	Y	Y	Y	U	Y	Y	Y	Y	Minor
Welsh Hospital Vending Welsh Assembly Government (2009)^78^	Y	U	U	U	U	U	U	No	Y	U	Major
East London Food for Life initiative Gray et al (2017)^79^	Y	Y	Y	Y	Y	U	Y	Y	Y	Y	Minor
“Healthful & Tasty: Sure” Sodium Reduction Trial Beer-Borst et al (2018)^80^; Beer-Borst et al (2019)^81^; Beer-Borst et al (2020)^82^	Y	Y	Y	Y	Y	U	Y	U	Y	Y	Moderate
Danish “6 a day” Worksite Canteen study Lassen et al (2004)^83^	Y	No	No	Y	Y	U	U	No	Y	Y	Major
New Zealand Heartbeat Program Young et al (2004)^84^	Y	Y	Y	Y	Y	Y	Y	U	Y	Y	Moderate

*Abbreviations:* U, unclear; Y, yes.

## DISCUSSION

The aim of this systematic literature review was to identify the barriers and facilitators to implementation of and compliance with healthy food and drink policies for the general adult population in public sector workplaces. Because food environments designed to enable healthy choices are a promising food service initiative,^85^ the results of this review will benefit all stakeholders involved in workplace health promotion. The findings indicate that the introduction of healthy food and drink policies is necessary to initiate and drive actions to make organizational food environments healthier, with the policy acting as a reference document and agreed upon standards. Studies suggest that healthy workplace food policies resulted in elimination of some unhealthy food and drink items. This signals that food environments in public sector workplaces, especially those in the health arena, often do not fully align with dietary guidelines prior to introduction of healthy food policies. Healthy food and drink policy as a workplace health promotion strategy has been gaining popularity,^86^ as illustrated by a recent review identifying 19 different nutrition policies for public institutions (excluding school settings) across 8 local jurisdictions in Australia.^87^ However, the results of this review indicate an increase in the prevalence of such policies does not guarantee full implementation and compliance, echoing the findings of a review of barriers and enablers to implementation in the school setting.^88^ Furthermore, the diversity of barriers, facilitators, and means to overcome challenges reported in the literature implies there is likely no 1 pathway to successful policy implementation. Certainly, adopting a long-term view with an ongoing and systemic change to overcome identified barriers and active efforts and engagement from various stakeholders (not only workplace food providers) are required for successful implementation.

Each of the 4 themes in this review refers directly or indirectly to the roles and responsibilities of local and national governments in influencing implementation of public workplace food policies. The most evident roles are developing a robust, mandatory, and monitored policy and providing financial and resource support for implementation, although funding should also encompass regular evaluation and monitoring^89^ that may be an undervalued facilitator in policy implementation, as highlighted previously in the review of Australian policies.^87^ Barriers to mandating government public nutrition policies generally stem from food industry pressure and lobbying, beliefs about personal responsibility for healthy eating and infringement on freedom, combined with a preference for voluntary food policies^90^ and prioritizing physical activity rather than healthy eating to prevent diet-related diseases.^91^ Yet, government-legislated policies, adopted by senior workplace management and written into food providers' contracts, are the only policies that can be legally enforced.^86^ However, the role of local and national government stakeholders extends to the introduction of other public health nutrition policies to synergistically support the implementation of healthy food and drink policies in public sector workplaces.

Mandatory national healthy reformulation targets for packaged foods can improve the overall food supply and make healthier policy-compliant options more feasible for food providers to source, as evident in this review. Targets also encourage such products to be generally more acceptable by the public^92^ while increasing customer demand,^93^ which could assist in overcoming the identified barrier of customer resistance to healthier options. Additional avenues of indirectly incentivizing private sector manufacturers to develop new healthier products or reformulate existing ones include front-of-pack and warning nutrition labels and taxation on unhealthy nutrients.^94^ However, the adoption of consistent mandatory nutrition standards across all public institutions, such as healthcare facilities, government agencies, recreation centers, and schools within national jurisdictions, is likely to create significant market demand, thus also incentivizing product reformulation and possibly mitigating some of the supply-related barriers specified in this review. Furthermore, consistency in nutritional criteria for different types of public sector workplaces could allow food supply-chain stakeholders to operate at scale across national regions.

Legislative action may also influence customer demand through zoning regulations for unhealthy food outlets in the vicinity of public sector institutions.^95^ The loss of customers to nearby competitors that were not covered by the same policy criteria was identified as a significant barrier in this review and has similarly been noted in school settings.^88^ However, adopting zoning policies often requires a long-term view, considering zoning regulations usually affect opening of new food outlets, rather than limiting existing food outlets selling unhealthy options.^96^ In the interim, senior workplace management could collaborate with nearby food providers to reduce their unhealthy food offerings in line with an adopted policy,^97^ supported by local government’s financial incentives for voluntary compliance.^95^ Evaluation of the local government–led Healthier Catering Commitment in London indicated high resource and time needs to engage with food operators.^98^ Yet, such initiatives may be imperative to sustain economic viability of workplace food providers. Furthermore, providing monetary awards or incentives^95^^,^^99^ or reducing payable lease or commission rates for compliant food outlets within a workplace may also mitigate profit loss after implementation, as evident from sales data analysis in some studies.^34^^,^^78^

At the individual workplace food-outlet level, findings from this review indicate implementation support personnel and food providers should focus on minimizing costs associated with implementation while optimizing business operations to maximize profits. Although direct financial support will assist food providers in minimizing costs, development and provision of relevant tools and resources is also an important element of an enabling implementation plan. The WHO suggests technical assistance and supporting materials can include administrative aids, policy-specific training, compliant-product and -supplier lists, sample recipes and menus, and compliance-monitoring tools,^14^ similar to the facilitating tools and resources identified in this review. The Healthy Eating Advisory Service, funded by the Victorian Government in Australia, is an example of support provided by experienced nutritionists and dietitians for implementation of healthy food and drink public sector policies.^100^ Such centralized, free, and accessible toolkits may decrease individual workplace and food providers’ operational and labor costs associated with finding new compliant products and suppliers and developing healthy recipes and staff training, while providing a communication platform for supply-side stakeholders. The resources could also suggest additional revenue stream ideas of nonfood-based merchandise or fundraising options to offset profit loss associated with elimination of unhealthy products.

Results of this review suggest removal of familiar unhealthy food and drink, rather than addition of healthier options, induces customer dissatisfaction associated with implementation, although a gradual approach to changes and frequent communication with consumers may assist with overcoming this barrier. However, to facilitate customer demand for compliant foods and drinks, and maximize workplace food providers’ profits, the focus should be placed on the main drivers behind customers’ motivation: taste and price.^12^ The common association of healthy foods as unpleasant and unhealthy foods as tasty^101^ suggests strategies such as naming vegetable dishes figuratively using word play, rather than literally,^102^ could influence customers’ acceptance and anticipatory enjoyment of healthier options, especially when foods are presented in an appetizing manner.^12^ Furthermore, results of this review indicate that letting customers try new products and gathering feedback on the samples can also increase customers’ support for implementation while guiding food providers to meet market product requirements. A complementary strategy of increasing the price of unhealthy products while decreasing price of healthy products^103–105^ could also manifest compliant foods as more desirable and influence customers’ choice while mitigating food providers’ fears of profit loss. Additional strategies outlined in this review include loyalty programs and meal deals aimed at compliant items only. This review’s findings suggest that removing unhealthy options as the first step in implementation is unlikely to create instant customer demand for healthy compliant options. However, pricing strategies could habituate customers to healthier options being better value for money while gradually decreasing the availability of and demand for unhealthy foods and drinks, resulting in healthy choices becoming the preferred choice^94^ in public sector workplaces.

### Characteristics and quality of the evidence

As expected, there were variations in the design and methods used across the 41 studies included in this review, because no restrictions were placed on methodological aspects and because identification of barriers and facilitators to implementation is often integrated into more-comprehensive evaluations of healthy food and drink policies. Interviews were the predominant method used to gather information on barriers and facilitators, followed by surveys and questionnaires. Studies that included a large number of participants tended to use self-reported surveys and focus groups as compared with smaller studies, which used mostly interviews. However, a large sample size does not necessarily provide higher confidence in study findings, because qualitative research aims for depth rather than breadth of findings and insights, and this can be achieved with a sample size of only a few purposively selected informed participants.^106^ The generally poor descriptions of data collection and analysis methods in the studies have been previously identified as a limitation in generic qualitative research.^107^ Nonetheless, we are confident in the review’s findings because the emphasis was placed on answering the review’s question, which all studies have contributed to, rather than their research design and methodology.^29^

### Strengths and limitations of the review

Several strengths of this systematic review should be noted. We used a systematic approach to identify relevant studies and reports, using a comprehensive database search strategy developed with a research librarian, as well as a broad search for grey literature reports. Additionally, the automated search update of the main scientific databases up until the data extraction stage resulted in the inclusion of 1 additional study published after the main searches were conducted. All included studies were assessed for their methodological strengths and limitations using a single tool that allowed us to assess and demonstrate confidence in the review’s findings. The development and interpretation of findings followed a widely accepted rigorous meta-synthesis approach guided by an author experienced in qualitative studies, resulting in analytic themes that did not simply repeat the data reported in the primary studies but synthesized a large number of studies spanning different policies, years, settings, and countries. Furthermore, this review was completed by a team of researchers with nutrition and food service backgrounds and experienced in developing and evaluating healthy food and drink policies, systematic reviews, and qualitative research methods.

One limitation of this review was restriction of eligible studies to those published in English, which has likely restricted study locations to English-speaking countries. Nevertheless, studies from the Netherlands, Denmark, and South Korea (countries where English is not an official language) were published in English and included in this review. Additionally, although the search strategy identified a substantial number of studies eligible for inclusion in this review, it is possible some relevant studies may have been missed in the search. Another limitation in this review was the synthesis of data from primary studies that were published in many different formats, mainly relying on authors’ analysis and interpretation of their raw data. It is unknown whether authors reported all their data in their findings or if they may have omitted data they deemed irrelevant.

### Implications for policy and practice

Political will from national and local governments is required to enable and facilitate implementation of healthy food and drink policies and compliance in public sector workplaces. Policy makers should ensure healthy food and drink policies are mandatory, able to be legally endorsed, equally applicable to all food operators, and feasible to implement. Government bodies also must consider the requirements for and provide financial and resource support for implementation, which may take the form of subsidies, monetary incentives, dedicated staff, and appropriate tools and resources. To further support implementation, governments can introduce complementary legislative nutrition policies mentioned in the discussion to influence overall national food supply and demand for healthier options.

Implementation at a workplace level must be based on open and regular communication between all stakeholders involved in and affected by the implementation of the healthy food and drink policy. Each workplace should develop an enabling and feasible implementation plan based on available resources and capacity to change. Food providers, support personnel, and senior workplace management should also collaborate prior to adoption and continually during the implementation to focus on and put effort into increasing customer demand for healthier options by using strategies outlined in this review.

### Research implications

This review’s findings suggest additional directions for research on healthy food and drink policies in public sector workplaces. First, the overall poor undertaking and description of data collection and analysis methods highlight the need for better-quality research and reporting practice of qualitative methods. Second, because only 1 study in this review (in Washington state in the United States) undertook repeated yearly evaluations of the adopted guidelines over a 5-year period, more well-designed longitudinal research of the implementation and impact of such policies, including the financial impact on the food providers, is needed in public sector institutions. Third, research efforts should focus on identifying suitable strategies to increase customer demand and influence their choice toward healthier options in public sector institutions. Last, auditing current supporting tools and resources and evaluating their impact on facilitating implementation of healthy food and drink policies are needed to determine tools and resources necessary for successful policy implementation.

## CONCLUSION

The increasing prevalence of nutrition policies aimed at regulating public sector food environments emphasizes recognition of the importance the provision of foods and drinks has on individual choice. However, this review indicates that food providers and supporting implementation staff are likely to face multiple barriers when implementing food and drink policies in public sector workplaces. Because barriers are unlikely to resolve on their own, focusing on facilitating elements identified in this review, and active and collaborative effort from all stakeholders involved in implementation, could mitigate challenges arising from adoption of such policies. In summary, policy implementation should consider the availability, acceptance, and demand for healthier compliant options, food providers’ readiness to change, and financial and resource availability. Thus, understanding barriers and facilitators to successful policy implementation will significantly benefit stakeholders interested in, or engaging in, healthy food and drink policy development and implementation.

## Supplementary Material

nuad062_Supplementary_Data

## Supporting Information

The following Supporting Information is available through the online version of this article at the publisher’s website.

*Appendix S1* **PRISMA checklist**

*Appendix S2* **Search log and results**

*Appendix S3* **Key findings from included studies**

*Data availability.* All data used in this review were extracted from published literature. All data sets generated or analyzed during this study are included in this published article and its supporting online files.
