# Supplementary material for: Barriers and facilitators to implementation of healthy food and drink policies in public sector workplaces: a systematic literature review
Source: Nutr Rev. 2023 Jun 19;82(4):503–35. doi: 10.1093/nutrit/nuad062 (PMC10925903; doi:10.1093/nutrit/nuad062)
Supplement: nuad062_Supplementary_Data [file nuad062_supplementary_data.zip › nuad062_Supplementary_Data/Appendix S2 Search log and results.pdf]

Appendix S2 Search log and results of the initial databases search

Database: **Ovid MEDLINE(R) Epub Ahead of Print, In Process & Other Non-Indexed Citations, Ovid MEDLINE (R) Daily, and Ovid MEDLINE (R) 1946-Present**

Date of search: **7 April 2021**

#	Search terms	Results
1	Workplace/	24281
2	(work environment* or workplace? or work place? or worksite? or work site? or employee* or staff or worker*).ti,ab,kw,kf.	407022
3	Local Government/	3423
4	State Government/	11983
5	Public Facilities/	1260
6	exp "Sports and Recreational Facilities"/	5355
7	((city or cities or local government* or state government* or city government* or township government* or town government* or local council* or state council* or city council* or town council* or local or public or publicly?funded or publically?funded or council?owned or government?owned or city?owned or town?owned or health or health care or healthcare) adj3 (service? or facility or facilities or office? or building? or sport? club? or club? or swimming pool? or recreation* or park or parks)).ti,ab,kw,kf.	222031
8	(hospital or hospitals).ti,ab,kw,kf.	1212972
9	Universities/	43162
10	(university* or universities or campus*).ti,ab,kw,kf.	391068
11	or/1-10	1951233
12	Food/	34070
13	Beverages/	15358
14	(food? or beverage? or drink? or meal? or snack? or snacking or menu? or menu planning or soft drink? or soda? or confectionary or sweetened drink? or sweetened beverage? or carbonated drink? or carbonated beverage? or nutritio* or diet*).ti,ab,kw,kf.	1251585
15	Diet, Healthy/	4722
16	(health* food? or health* drink? or "health* food? and drink?").ti,ab,kw,kf.	7774
17	or/12-16	1264309
18	(food environment? or beverage environment? or drink environment? or eating environment?).ti,ab,kw,kf.	2857
19	Food Dispensers, Automatic/	351
20	(food? dispens* or drink? dispens* or snack? dispens* or vendor? or vending or vending machine? or supply).ti,ab,kw,kf.	162182
21	Food Services/	5746
22	(food service* or foodservice* or catering or caterer* or canteen* or cafeteria* or food outlet* or food retail* or retail* or food kiosk? or food shop? or lunchroom? or dining).ti,ab,kw,kf.	21240
23	Hospital shops/	374

24	or/18-23	188103
25	Nutrition Policy/	9711
26	policy/	3198
27	organizational policy/	14312
28	(policy or policies or guideline* or criteria or strategy or strategies or standard or standards or regulation or regulations or initiative* or programme? or program? or intervention? or promotion? or promoting or procurement?).ti,ab,kw,kf.	5466094
29	Serving Size/	115
30	Portion Size/	536
31	(serving? size? or portion? size? or meal? size?).ti,ab,kw,kf.	3769
32	st.fs.	743496
33	or/25-32	5946021
34	11 and 17 and 24 and 33	3263
35	limit 34 to yr="2000 -Current"	**2456**

Database: **Ovid Embase (1980 to 2021 April 05)**

Date of search: **7 April 2021**

#	Search terms	Results
1	workplace/	45123
2	(work environment* or workplace? or work place? or worksite? or work site? or employee* or staff or worker*).ti,ab,kw.	508332
3	(local government? or state government?).ti,ab,kw.	8599
4	recreational park/	1076
5	(sport* facility or sport* facilities or recreational facility or recreational facilities).ti,ab,kw.	853
6	((city or cities or local government* or state government* or city government* or township government* or town government* or local council* or state council* or city council* or town council* or local or public or publicly?funded or publically?funded or council?owned or government?owned or city?owned or town?owned or health or health care or healthcare) adj3 (service? or facility or facilities or office? or building? or sport? club? or club? or swimming pool? or recreation* or park or parks)).ti,ab,kw.	260285
7	(hospital or hospitals).ti,ab,kw.	1795218
8	university/	111929
9	(university* or universities or campus*).ti,ab,kw.	680694
10	or/1-9	2792639
11	food/	69825
12	beverage/	18874
13	(food? or beverage? or drink? or meal? or snack? or snacking or menu? or menu planning or soft drink? or soda? or confectionary or sweetened drink? or sweetened	1507558

	beverage? or carbonated drink? or carbonated beverage? or nutritio* or diet*).ti,ab,kw.	
14	healthy diet/	4416
15	(health* food? or health* drink? or "health* food? and drink?").ti,ab,kw.	10164
16	or/11-15	1519199
17	catering service/	18234
18	(food environment? or beverage environment? or drink environment? or eating environment?).ti,ab,kw.	3522
19	food handling/	21245
20	(food? dispens* or drink? dispens* or snack? dispens* or vendor? or vending or vending machine? or supply).ti,ab,kw.	181130
21	(food service* or foodservice* or catering or caterer* or canteen* or cafeteria* or food outlet* or food retail* or retail* or food kiosk? or food shop? or lunchroom? or dining).ti,ab,kw.	24392
22	cafeteria diet/	179
23	or/17-22	237474
24	nutrition policy/	1686
25	policy/	87912
26	organizational policy/	1525
27	(policy or policies or guideline* or criteria or strategy or strategies or standard or standards or regulation or regulations or initiative* or programme? or program? or intervention? or promotion? or promoting or procurement?).ti,ab,kw.	7308065
28	portion size/	2454
29	meal size/	192
30	food quantity/	82
31	(serving? size? or portion? size? or meal? size?).ti,ab,kw.	4932
32	st.fs.	0
33	or/24-32	7345241
34	10 and 16 and 23 and 33	4394
35	limit 34 to yr="2000 -Current"	**3940**

Database: **Scopus**
Date of search: **7 April 2021**

Search terms	Results
(TITLE-ABS-KEY ("work environment*" OR workplace* OR "work place*" OR worksite* OR "work site*" OR employee* OR staff OR worker* OR "local government*" OR "state government*" OR "public facility" OR "public facilities" OR "recreational facility" OR "recreational facilities" OR hospital OR hospitals OR university OR universities OR campus* OR (city OR cities OR "local government*" OR "state government*" OR "city government*" OR "township government*" OR "town government*" OR "local council*" OR "state council*" OR "city council*" OR "town council*" OR local OR public OR "publicly funded" OR	

"publically funded" OR "council owned" OR "government owned" OR "city owned" OR "town owned" OR health OR "health care" OR healthcare) W/3 (service* OR facility OR facilities OR office* OR building* OR "sport? club?" OR club? OR "swimming pool?" OR recreation* OR park OR parks))	
AND TITLE-ABS-KEY (food? OR beverage? OR drink? OR meal? OR snack? OR snacking OR menu? OR "menu planning" OR "soft drink?" OR soda? OR confectionary OR "sweetened drink?" OR "sweetened beverage?" OR "carbonated drink?" OR "carbonated beverage?" OR nutritio* OR diet* OR "health* food?" OR "health* drink?" OR {healthy food and drink})	
AND TITLE-ABS-KEY ("food environment?" OR "beverage environment?" OR "drink environment?" OR "eating environment?" OR "food? dispens*" OR "drink? dispens*" OR "snack? dispens*" OR vendor? OR vending OR "vending machine?" OR supply OR "food service*" OR foodservice* OR catering OR caterer* OR canteen* OR cafeteria* OR "food outlet*" OR "food retail*" OR retail* OR "food kiosk?" OR "food shop?" OR lunchroom* OR dining OR "hospital shop*")	
AND TITLE-ABS-KEY ("nutrition polic*" OR "organi?ational polic*" OR policy OR policies OR guideline* OR criteria OR strategy OR strategies OR standard OR standards OR regulation OR regulations OR initiative* OR programme? OR program? OR intervention? OR promotion? OR promoting OR procurement? OR "serving? size?" OR "portion? size?" OR "meal? size?"))	3527
AND (LIMIT-TO (PUBYEAR , 2021) OR LIMIT-TO (PUBYEAR , 2020) OR LIMIT-TO (PUBYEAR , 2019) OR LIMIT-TO (PUBYEAR , 2018) OR LIMIT-TO (PUBYEAR , 2017) OR LIMIT-TO (PUBYEAR , 2016) OR LIMIT-TO (PUBYEAR , 2015) OR LIMIT-TO (PUBYEAR , 2014) OR LIMIT-TO (PUBYEAR , 2013) OR LIMIT-TO (PUBYEAR , 2012) OR LIMIT-TO (PUBYEAR , 2011) OR LIMIT-TO (PUBYEAR , 2010) OR LIMIT-TO (PUBYEAR , 2009) OR LIMIT-TO (PUBYEAR , 2008) OR LIMIT-TO (PUBYEAR , 2007) OR LIMIT-TO (PUBYEAR , 2006) OR LIMIT-TO (PUBYEAR , 2005) OR LIMIT-TO (PUBYEAR , 2004) OR LIMIT-TO (PUBYEAR , 2003) OR LIMIT-TO (PUBYEAR , 2002) OR LIMIT-TO (PUBYEAR , 2001) OR LIMIT-TO (PUBYEAR , 2000))	**2440**

Database: **Cumulative Index to Nursing and Allied Health Literature (CINAHL Plus, EBSCOhost)**

Date of search: **7 April 2021**

#	Search terms	Results
S30	S10 AND S15 AND S21 AND S28 Limiters - Publication Year: 2000-2021	**1,592**
S29	S10 AND S15 AND S21 AND S28	1,690
S28	S22 OR S23 OR S24 OR S25 OR S26 OR S27	1,668,088
S27	MW "st"	196,776
S26	TI ("serving# size#" or "portion# size#" or "meal# size#") OR AB ("serving# size#" or "portion# size#" or "meal# size#")	1,466
S25	(MH "Portion Size")	1,860
S24	TI (policy or policies or guideline* or criteria or strategy or strategies or standard or standards or regulation or regulations or initiative* or programme# or program#	1,537,054

	or intervention# or promotion# or promoting or procurement#) OR AB (policy or policies or guideline* or criteria or strategy or strategies or standard or standards or regulation or regulations or initiative* or programme# or program# or intervention# or promotion# or promoting or procurement#)	
S23	(MH "Organizational Policies")	13,362
S22	(MH "Nutrition Policy")	4,499
S21	S16 OR S17 OR S18 OR S19 OR S20	45,926
S20	TI ("food service*" or foodservice* or catering or caterer* or canteen* or cafeteria* or "food outlet*" or "food retail*" or retail* or "food kiosk#" or "food shop#" or lunchroom# or dining) OR AB (("food service*" or foodservice* or catering or caterer* or canteen* or cafeteria* or "food outlet*" or "food retail*" or retail* or "food kiosk#" or "food shop#" or lunchroom# or dining)	8,866
S19	(MH "Food Services")	7,137
S18	TI ("food# dispens*" or "drink# dispens*" or "snack# dispens*" or vendor# or vending or "vending machine#" or supply) OR AB ("food# dispens*" or "drink# dispens*" or "snack# dispens*" or vendor# or vending or "vending machine#" or supply)	30,875
S17	(MH "Food Dispensers, Automatic")	135
S16	TI ("food environment#" or "beverage environment#" or "drink environment#" or "eating environment#") OR AB ("food environment#" or "beverage environment#" or "drink environment#" or "eating environment#")	1,585
S15	S11 OR S12 OR S13 OR S14	315,145
S14	TI ("health* food#" or "health* drink#" or "health* food# and drink#") OR AB ("health* food#" or "health* drink#" or "health* food# and drink#")	4,719
S13	TI (food# or beverage# or drink# or meal# or snack# or snacking or menu# or "menu planning" or "soft drink#" or soda# or confectionary or "sweetened drink#" or "sweetened beverage#" or "carbonated drink#" or "carbonated beverage#" or nutritio* or diet*) OR AB (food# or beverage# or drink# or meal# or snack# or snacking or menu# or "menu planning" or "soft drink#" or soda# or confectionary or "sweetened drink#" or "sweetened beverage#" or "carbonated drink#" or "carbonated beverage#" or nutritio* or diet*)	308,326
S12	(MH "Beverages")	6,516
S11	(MH "Food")	14,862
S10	S1 OR S2 OR S3 OR S4 OR S5 OR S6 OR S7 OR S8 OR S9	838,423
S9	TI (university* or universities or campus*) OR AB (university* or universities or campus*)	152,489
S8	(MH "Colleges and Universities")	26,712
S7	TI (hospital or hospitals) OR AB (hospital or hospitals)	420,302
S6	TI ((city or cities or "local government*" or "state government*" or "city government*" or "township government*" or "town government*" or "local council*" or "state council*" or "city council*" or "town council*" or local or public or "publicly#funded" or "publically#funded" or "council#owned" or "government#owned" or "city#owned" or "town#owned" or health or "health care" or healthcare) N3 (service* or facility or facilities or office# or building# or "sport* club*" or club# or "swimming pool#" or recreation* or park or parks)) OR AB ((city or cities or "local government*" or "state government*" or "city government*" or "township government*" or "town government*" or "local council*" or "state council*" or "city council*" or "town council*" or local or public or "publicly#funded" or "publically#funded" or "council#owned" or	131,262

	"government#owned" or "city#owned" or "town#owned" or health or "health care" or healthcare) N3 (service* or facility or facilities or office# or building# or "sport* club*" or club# or "swimming pool#" or recreation* or park or parks))	
S5	(MH "Sports Facilities")	193
S4	(MH "Public Facilities")	776
S3	(MH "Federal Government") OR (MH "Local Government") OR (MH "State Government")	5,812
S2	TI (("work environment*" or workplace# or "work place#" or worksite# or "work site#" or employee* or staff or worker*)) OR AB (("work environment*" or workplace# or "work place#" or worksite# or "work site#" or employee* or staff or worker*))	234,609
S1	(MH "Work Environment") OR "Workplace*"	58,273

Database: **Cochrane Central Register of Controlled Trials (CENTRAL)**
Date of search: **7 April 2021**

#	Search terms	Results
1	[mh ^"workplace"]	855
2	("work environment*" or workplace? or "work place?" or worksite? or "work site?" or employee* or staff or worker*):ti,ab,kw	33582
3	[mh ^"Local Government"]	15
4	[mh ^"State Government"]	11
5	[mh ^"Public Facilities"]	18
6	[mh "Sports and Recreational Facilities"]	116
7	((city or cities or "local government*" or "state government*" or "city government*" or "township government*" or "town government*" or "local council*" or "state council*" or "city council*" or "town council*" or local or public or "publicly?funded" or "publically?funded" or "council?owned" or "government?owned" or "city?owned" or "town?owned" or health or "health care" or healthcare) NEAR/3 (service? or facility or facilities or office? or building? or "sport? club?" or club? or "swimming pool?" or recreation* or park or parks)):ti,ab,kw	26125
8	(hospital or hospitals):ti,ab,kw	154391
9	[mh ^Universities]	940
10	(university* or universities or campus*):ti,ab,kw	53356
11	{or #1-#10}	221632
12	[mh ^Food]	1289
13	[mh ^Beverages]	1749
14	(food? or beverage? or drink? or meal? or snack? or snacking or menu? or "menu planning" or "soft drink?" or soda? or confectionary or "sweetened drink?" or "sweetened beverage?" or "carbonated drink?" or "carbonated beverage?" or nutritio* or diet*):ti,ab,kw	158800
15	[mh ^"Diet, Healthy"]	496
16	("health* food?" or "health* drink?" or "health* food? and drink?"):ti,ab,kw	164

17	{or #12-#16}	158800
18	("food environment?" or "beverage environment?" or "drink environment?" or "eating environment?"):ti,ab,kw	173
19	[mh ^"Food Dispensers, Automatic"]	11
20	("food? dispens*" or "drink? dispens*" or "snack? dispens*" or vendor? or vending or "vending machine?" or supply):ti,ab,kw	14213
21	[mh ^"Food Services"]	258
22	("food service*" or foodservice* or catering or caterer* or canteen* or cafeteria* or "food outlet*" or "food retail*" or retail* or "food kiosk?" or "food shop?" or lunchroom* or dining):ti,ab,kw	1083
23	[mh ^"Hospital shops"]	1
24	{or #18-#23}	15478
25	[mh ^"Nutrition Policy"]	334
26	[mh ^policy]	16
27	[mh ^"organizational policy"]	95
28	(policy or policies or guideline* or criteria or strategy or strategies or standard or standards or regulation or regulations or initiative* or programme? or program? or intervention? or promotion? or promoting or procurement?):ti,ab,kw	708351
29	[mh ^"Serving Size"]	14
30	[mh ^"Portion Size"]	96
31	("serving? size?" or "portion? size?" or "meal? size?"):ti,ab,kw	644
32	st.fs	1
33	{or #25-#32}	708594
34	{and #11, #17, #24, #33} with Cochrane Library publication date Between Jan 2000 and Apr 2021	**704**

Database: **APA PsycInfo (1806 to March Week 5 2021)**
Date of search: **7 April 2021**

#	Search terms	Results
1	workplace intervention/	996
2	("work environment*" or workplace? or "work place?" or worksite? or "work site?" or employee* or staff or worker*).ti,ab.	248207
3	facilities/	1142
4	recreation areas/	1110
5	((city or cities or "local government*" or "state government*" or "city government*" or "township government*" or "town government*" or "local council*" or "state council*" or "city council*" or "town council*" or local or public or "publicly?funded" or "publically?funded" or "council?owned" or "government?owned" or "city?owned" or "town?owned" or health or "health care" or healthcare) adj3 (service? or facility or facilities or office? or building? or "sport? club?" or club? or "swimming pool?" or recreation* or park or parks)).ti,ab.	79156
6	(hospital or hospitals).ti,ab.	126383

7	colleges/ or community colleges/ or college environment/ or higher education/ or military schools/	35095
8	(university* or universities or campus*).ti,ab.	173859
9	1 or 2 or 3 or 4 or 5 or 6 or 7 or 8	579819
10	food/	14288
11	"beverages (nonalcoholic)"/	1702
12	(food? or beverage? or drink? or meal? or snack? or snacking or menu? or "menu planning" or "soft drink?" or soda? or confectionary or "sweetened drink?" or "sweetened beverage?" or "carbonated drink?" or "carbonated beverage?" or nutritio* or diet*).ti,ab.	148749
13	("health* food?" or "health* drink?" or "health* food? and drink?").ti,ab.	2361
14	10 or 11 or 12 or 13	150024
15	("food environment?" or "beverage environment?" or "drink environment?" or "eating environment?").ti,ab.	954
16	("food? dispens*" or "drink? dispens*" or "snack? dispens*" or vendor? or vending or "vending machine?" or supply).ti,ab.	16559
17	("food service*" or foodservice* or catering or caterer* or canteen* or cafeteria* or "food outlet*" or "food retail*" or retail* or "food kiosk?" or "food shop?" or lunchroom? or dining or "hospital shop?").ti,ab.	11978
18	15 or 16 or 17	28481
19	policy making/	23081
20	("nutrition* policy" or "nutrition* policies" or policy or policies or guideline* or criteria or strategy or strategies or standard or standards or regulation or regulations or initiative* or programme? or program? or intervention? or promotion? or promoting or procurement?).ti,ab.	1405935
21	("serving? size?" or "portion? size?" or "meal? size?").ti,ab.	1256
22	19 or 20 or 21	1408243
23	9 and 14 and 18 and 22	672
24	limit 23 to yr="2000 -Current"	**620**

Database: **PubMed (1946 to present, updated daily)**

Date of search: **7 April 2021**

#	Search terms	Results
35	#34 AND 2000:2022[dp]	**1,832**
34	#11 AND #17 AND #24 AND #33	2,293
33	#25 OR #26 OR #27 OR #28 OR #29 OR #30 OR #31 OR #32	5,051,522
32	st.fs.	9
31	("serving size"[tiab] OR "serving sizes"[tiab] OR "servings size"[tiab] OR "servings sizes"[tiab] OR "portion size"[tiab] OR "portion sizes"[tiab] OR "portions size"[tiab] OR "portions sizes"[tiab] OR "meal size"[tiab] OR "meal sizes"[tiab] OR "meals size"[tiab] OR "meals sizes"[tiab])	3,753
30	Portion Size[mesh:noexp]	537

29	Serving Size[mesh:noexp]	115
28	(policy[tiab] OR policies[tiab] OR guideline*[tiab] OR criteria[tiab] OR strategy[tiab] OR strategies[tiab] OR standard[tiab] OR standards[tiab] OR regulation[tiab] OR regulations[tiab] OR initiative*[tiab] OR programme?[tiab] OR program?[tiab] OR intervention?[tiab] OR promotion?[tiab] OR promoting[tiab] OR procurement?[tiab])	5,037,171
27	organizational policy[mesh:noexp]	14,314
26	policy[mesh:noexp]	3,201
25	Nutrition Policy[mesh:noexp]	9,715
24	#18 OR #19 OR #20 OR #21 OR #22 OR #23	184,547
23	Hospital shops[mesh:noexp]	374
22	("food service"[tiab] OR "food services"[tiab] OR foodservice*[tiab] OR catering[tiab] OR caterer*[tiab] OR canteen*[tiab] OR cafeteria*[tiab] OR "food outlet"[tiab] OR "food outlets"[tiab] OR "food retail"[tiab] OR "food retails"[tiab] OR retail*[tiab] OR "food kiosk"[tiab] OR "food kiosks" OR "food shop"[tiab] OR "food shops"[tiab] OR lunchroom*[tiab] OR dining[tiab])	21,243
21	Food Services[mesh:noexp]	5,746
20	("food dispenser"[tiab] OR "food dispensing"[tiab] OR "food dispensers"[tiab] OR "drink dispenser"[tiab] OR "drink dispensing"[tiab] OR "drink dispensers"[tiab] OR "snack dispenser"[tiab] OR "snack dispensing"[tiab] OR "snack dispensers"[tiab] OR vendor?[tiab] OR vending[tiab] OR "vending machine"[tiab] OR "vending machines"[tiab] OR supply[tiab])	158,429
19	Food Dispensers, Automatic[mesh:noexp]	351
18	("food environment"[tiab] OR "food environments"[tiab] OR "beverage environment"[tiab] OR "beverage environments"[tiab] OR "drink environment"[tiab] OR "drink environments"[tiab] OR "eating environment"[tiab] OR "eating environments"[tiab])	2,848
17	#12 OR #13 OR #14 OR #15 OR #16	1,226,954
16	("healthy food"[tiab] OR "healthy foods"[tiab] OR "healthier food"[tiab] OR "healthier foods"[tiab] OR "healthy drink"[tiab] OR "healthy drinks"[tiab] OR "healthier drink"[tiab] OR "healthier drinks"[tiab] OR "healthy food and drink"[tiab] OR "healthier food and drink"[tiab] OR "healthy foods and drinks"[tiab] OR "healthier foods and drinks"[tiab])	5,340
15	Diet, Healthy[mesh:noexp]	4,739
14	(food?[tiab] OR beverage?[tiab] OR drink?[tiab] OR meal?[tiab] OR snack?[tiab] OR snacking[tiab] OR menu?[tiab] OR "menu planning"[tiab] OR "soft drink"[tiab] OR "soft drinks"[tiab] OR soda?[tiab] OR confectionary[tiab] OR "sweetened drink"[tiab] OR "sweetened drinks"[tiab] OR "sweetened beverage"[tiab] OR "sweetened beverages"[tiab] OR "carbonated drink"[tiab] OR "carbonated drinks"[tiab] OR "carbonated beverage"[tiab] OR "carbonated beverages"[tiab] OR nutritio*[tiab] OR diet*[tiab])	1,211,617
13	Beverages[mesh:noexp]	15,362
12	Food[mesh:noexp]	34,079
11	#1 OR #2 OR #3 OR #4 OR #5 OR #6 OR #7 OR #8 OR #9 OR #10	1,802,049
10	(university[tiab] OR universities[tiab] OR campus*[tiab])	391,038
9	Universities[mesh:noexp]	43,180
8	(hospital[tiab] OR hospitals[tiab])	1,212,931

7	((city[tiab] OR cities[tiab] OR "local government"[tiab] OR "local governments"[tiab] OR "state government"[tiab] OR "state governments"[tiab] OR "city government"[tiab] OR "city governments"[tiab] OR "township government"[tiab] OR "township governments"[tiab] OR "town government"[tiab] OR "town governments"[tiab] OR "local council"[tiab] OR "local councils"[tiab] OR "state council"[tiab] OR "state councils"[tiab] OR "city council"[tiab] OR "city councils"[tiab] OR "town council"[tiab] OR "town councils"[tiab] OR local[tiab] OR public[tiab] OR publicly?funded[tiab] OR publically?funded[tiab] OR council?owned[tiab] OR government?owned[tiab] OR city?owned[tiab] OR town?owned[tiab] OR health[tiab] OR "health care"[tiab] OR healthcare[tiab]) n3 (service?[tiab] OR facility[tiab] OR facilities[tiab] OR office?[tiab] OR building?[tiab] OR "sport club"[tiab] OR "sports club"[tiab] OR "sport clubs"[tiab] OR "sports clubs"[tiab] OR club?[tiab] OR "swimming pool"[tiab] OR "swimming pools"[tiab] OR recreation*[tiab] OR park[tiab] OR parks[tiab]))	847
6	Sports and Recreational Facilities[mesh]	5,368
5	Public Facilities[mesh:noexp]	1,261
4	State Government[mesh:noexp]	11,986
3	Local Government[mesh:noexp]	3,424
2	("work environment"[tiab] OR "work environments"[tiab] OR workplace?[tiab] OR "work place"[tiab] OR "work places"[tiab] OR worksite?[tiab] OR "work site"[tiab] OR "work sites"[tiab] OR employee*[tiab] OR staff[tiab] OR worker*[tiab])	405,154
1	Workplace[mesh:noexp]	24,291

Database: **Google Scholar (limit to first 200 results)** (https://scholar.google.com)
Date of search: **7 April 2021**

Search terms	Results
("work environment*" OR workplace* OR "work place*" OR worksite* OR "work site*" OR employee* OR staff OR worker* OR "local government*" OR "state government*" OR "public facility" OR "public facilities" OR "recreational facility" OR "recreational facilities" OR hospital OR hospitals OR university OR universities OR campus* OR (city OR cities OR "local government*" OR "state government*" OR "city government*" OR "township government*" OR "town government*" OR "local council*" OR "state council*" OR "city council*" OR "town council*" OR local OR public OR "publicly funded" OR "publically funded" OR "council owned" OR "government owned" OR "city owned" OR "town owned" OR health OR "health care" OR healthcare) AROUND(3) (service* OR facility OR facilities OR office* OR building* OR "sport* club*" OR club* OR "swimming pool*" OR recreation* OR park OR parks)) AND (food* OR beverage* OR drink* OR meal* OR snack* OR snacking OR menu* OR "menu planning" OR "soft drink*" OR soda* OR confectionary OR "sweetened drink*" OR "sweetened beverage*" OR "carbonated drink*" OR "carbonated beverage*" OR nutritio* OR diet* OR "health* food*" OR "health* drink*" OR "healthy food and drink") AND ("food environment*" OR "beverage environment*" OR "drink environment*" OR "eating environment*" OR "food* dispens*" OR "drink* dispens*" OR "snack* dispens*" OR vendor* OR vending OR "vending machine*" OR supply OR "food service*" OR foodservice* OR catering OR caterer* OR canteen* OR cafeteria* OR "food outlet*" OR "food retail*" OR retail* OR "food kiosk*" OR "food shop*" OR lunchroom* OR dining OR "hospital shop*") AND ("nutrition polic*" OR "organi*ational polic*" OR policy OR policies OR guideline* OR	**First 200**

criteria OR strategy OR strategies OR standard OR standards OR regulation OR regulations OR initiative* OR programme* OR program* OR intervention* OR promotion* OR promoting OR procurement* OR "serving* size*" OR "portion* size*" OR "meal* size*")

Database: **Epistemonikos** (www.epistemonikos.org)
Date of search: **7 April 2021**

Search terms	Results
(title:(workplace OR "work place" OR worksite OR "work site" OR employee* OR staff OR worker OR hospital OR hospitals OR university OR universities OR campus OR "local government" OR "state government") OR abstract:(workplace OR "work place" OR worksite OR "work site" OR employee* OR staff OR worker OR hospital OR hospitals OR university OR universities OR campus OR "local government" OR "state government")) AND (title:(food OR beverage OR drink OR meal OR snack OR menu OR soda OR confectionary OR nutrition) OR abstract:(food OR beverage OR drink OR meal OR menu OR snack OR soda OR confectionary OR nutrition)) AND (title:("food environment" OR dispenser OR "vending machine" OR "vending machines" OR supply OR "food service" OR catering OR canteen OR cafeteria OR outlet OR retail OR kiosk OR lunchroom) OR abstract:("food environment" OR dispenser OR "vending machine" OR "vending machines" OR supply OR "food service" OR catering OR canteen OR cafeteria OR outlet OR retail OR kiosk OR lunchroom)) AND (title:(policy OR policies OR guideline OR guidelines OR criteria OR strategy OR strategies OR standard OR standards OR regulation OR initiative OR programme OR program OR intervention OR promotion OR procurement OR "serving size" OR "portion size") OR abstract:(policy OR policies OR guideline OR guidelines OR criteria OR strategy OR strategies OR standard OR standards OR regulation OR initiative OR programme OR program OR intervention OR promotion OR procurement OR "serving size" OR "portion size"))	**42**

Database: **ProQuest Dissertations & Theses Global** (Citations from 1861, Abstracts from 1980, Full text PDFs from 1996)
Date of search: **7 April 2021**

Search terms	Results
(ab(("work environment" OR "work environmental" OR "work environments" OR workplace* OR "work place" OR "work placed" OR "work placements" OR "work places" OR worksite* OR "work site" OR "work sites" OR employee* OR staff OR worker* OR "local government" OR "local governmental" OR "local governments" OR "state government" OR "state governmental" OR "state governments" OR "public facility" OR "public facilities" OR "recreational facility" OR "recreational facilities" OR hospital OR hospitals OR university OR universities OR campus*) OR ti(("work environment" OR "work environmental" OR "work environments") OR workplace* OR ("work place" OR "work placed" OR "work placements" OR "work places") OR worksite* OR ("work site" OR "work sites") OR employee* OR staff OR worker* OR ("local government" OR "local governmental" OR "local governments") OR ("state government" OR "state	

governmental" OR "state governments") OR "public facility" OR "public facilities" OR "recreational facility" OR "recreational facilities" OR hospital OR hospitals OR university OR universities OR campus*))	
AND (ab(food? OR beverage? OR drink? OR meal? OR snack? OR snacking OR menu? OR "menu planning" OR "soft drink?" OR soda? OR confectionary OR "sweetened drink?" OR "sweetened beverage?" OR "carbonated drink?" OR "carbonated beverage?" OR nutritio* OR diet* OR "health* food?" OR "health* drink?" OR "healthy food and drink") OR ti(food? OR beverage? OR drink? OR meal? OR snack? OR snacking OR menu? OR "menu planning" OR "soft drink?" OR soda? OR confectionary OR "sweetened drink?" OR "sweetened beverage?" OR "carbonated drink?" OR "carbonated beverage?" OR nutritio* OR diet* OR "health* food?" OR "health* drink?" OR "healthy food and drink"))	
AND (ab(("food environment?" OR "beverage environment?" OR "drink environment?" OR "eating environment?" OR "food? dispens*" OR "drink? dispens*" OR "snack? dispens*" OR vendor? OR vending OR "vending machine?" OR supply OR "food service" OR foodservice* OR catering OR caterer* OR canteen* OR cafeteria* OR "food outlet" OR "food outlets" OR "food retail" OR "food retailer" OR "food retailers" OR "food retailing" OR retail* OR "food kiosk?" OR "food shop?" OR lunchroom* OR dining OR "hospital shop*") OR ti("food environment?" OR "beverage environment?" OR "drink environment?" OR "eating environment?" OR "food? dispens*" OR "drink? dispens*" OR "snack? dispens*" OR vendor? OR vending OR "vending machine?" OR supply OR ("food service") OR foodservice* OR catering OR caterer* OR canteen* OR cafeteria* OR ("food outlet" OR "food outlets") OR ("food retail" OR "food retailer" OR "food retailers" OR "food retailing") OR retail* OR "food kiosk?" OR "food shop?" OR lunchroom* OR dining OR "hospital shop*"))	
AND (ab(("nutrition policy" OR "organi?ational polic*" OR policy OR policies OR guideline* OR criteria OR strategy OR strategies OR standard OR standards OR regulation OR regulations OR initiative* OR programme? OR program? OR intervention? OR promotion? OR promoting OR procurement? OR "serving? size?" OR "portion? size?" OR "meal? size?") OR ti(("nutrition policy") OR "organi?ational polic*" OR policy OR policies OR guideline* OR criteria OR strategy OR strategies OR standard OR standards OR regulation OR regulations OR initiative* OR programme? OR program? OR intervention? OR promotion? OR promoting OR procurement? OR "serving? size?" OR "portion? size?" OR "meal? size?"))	
Narrowed by: Entered date: 2000-01-01 - 2021-04-07; Language: English	**664**

Database: **Open Grey** (www.opengrey.eu)

Date of search: **7 April 2021**

Search terms	Results
("work environment*" OR workplace* OR "work place*" OR worksite* OR "work site*" OR employee* OR staff OR worker* OR "local government*" OR "state government*" OR "public facility" OR "public facilities" OR "recreational facility" OR "recreational facilities" OR hospital OR hospitals OR university OR universities OR campus* OR (city OR cities OR "local government*" OR "state government*" OR "city government*" OR "township government*" OR "town government*" OR "local council*" OR "state council*" OR "city council*" OR "town council*" OR local OR public OR "publicly funded" OR "publically funded" OR "council owned" OR "government owned" OR "city	**104**

owned" OR "town owned" OR health OR "health care" OR healthcare) NEAR/3 (service* OR facility OR facilities OR office* OR building* OR "sport* club*" OR club* OR "swimming pool*" OR recreation* OR park OR parks)) AND (food* OR beverage* OR drink* OR meal* OR snack* OR snacking OR menu* OR "menu planning" OR "soft drink*" OR soda* OR confectionary OR "sweetened drink*" OR "sweetened beverage*" OR "carbonated drink*" OR "carbonated beverage*" OR nutritio* OR diet* OR "health* food*" OR "health* drink*" OR "healthy food and drink") AND ("food environment*" OR "beverage environment*" OR "drink environment*" OR "eating environment*" OR "food* dispens*" OR "drink* dispens*" OR "snack* dispens*" OR vendor* OR vending OR "vending machine*" OR supply OR "food service*" OR foodservice* OR catering OR caterer* OR canteen* OR cafeteria* OR "food outlet*" OR "food retail*" OR retail* OR "food kiosk*" OR "food shop*" OR lunchroom* OR dining OR "hospital shop*") AND ("nutrition polic*" OR "organi*ational polic*" OR policy OR policies OR guideline* OR criteria OR strategy OR strategies OR standard OR standards OR regulation OR regulations OR initiative* OR programme* OR program* OR intervention* OR promotion* OR promoting OR procurement* OR "serving* size*" OR "portion* size*" OR "meal* size*")

Results of hand-searches of the remaining Grey Literature databases

Database (website)	Date of search	Results
Eldis (www.eldis.org)	7 April 2021	**0**
Grey Literature Reports (www.greylit.org)	8 April 2021	**56**
BASE (www.base-search.net/Search/Advanced)	8 April 2021	**488**
WHO Institutional Repository for Information Sharing (IRIS) (https://apps.who.int/iris/)	8 April 2021	**45**
International Food Policy Research Institute (IFPRI) (https://library.ifpri.info)	8 April 2021	**0**
WCRF NOURISHING database (https://policydatabase.wcrf.org)	8 April 2021	**4**
OECD iLibrary (www.oecd-library.org/search/advancedsearch)	8 April 2021	**1**
Government Agency and NGO websites in key English-speaking countries	8 April 2021 – 5 July 2021	**8**
