# Supplementary material for: Barriers and facilitators to implementation of healthy food and drink policies in public sector workplaces: a systematic literature review
Source: Nutr Rev. 2023 Jun 19;82(4):503–35. doi: 10.1093/nutrit/nuad062 (PMC10925903; doi:10.1093/nutrit/nuad062)
Supplement: nuad062_Supplementary_Data [file nuad062_supplementary_data.zip › nuad062_Supplementary_Data/Appendix S3 Key findings from included studies.pdf]

Appendix S3 Summary of key findings from included studies

**Study, publications Location**	**Summary of key findings** (Theme and sub-theme names outlines in publications were retained where possible)
**Healthy Choices in Victorian Recreation Centres**<br><br>Blake et al. (2020)[1]<br><br>Melbourne, Victoria, Australia	Six main themes:<br>1. Changes in stakeholders attitudes (more engagement, positive attitudes) and commitment over time<br>2. Role of stakeholders in implementing and maintaining guidelines changed over time<br>3. Logistics and practical strategies to gradually implement the guidelines with support from dietitian and health advisory service<br>4. Healthy food advocacy, with stakeholders having nutrition background generally more interested in the community food environments roles in promoting health and wellbeing<br>5. Commercial viability, with a general anticipation of profit loss decreasing over time (especially at sites where profits were unaffected)<br>6. Maximising customer experience and support for changes in healthy drink availability

Study, publications Location	Summary of key findings (Theme and sub-theme names outlines in publications were retained where possible)
**Healthy Choices in Alfred Health** (2017[2] journal publication)<br><br>Boelsen-Robinson et al. (2017)[2];<br>Peeters et al. (2017)[3];<br>Boelsen-Robinson (2019)[4];<br><br>Victoria, Australia	Four themes related to the context of policy implementation<br>1. Strong executive-level support<br>1.1 Appointment of health promotion manager<br>1.2 Hands-on approach of senior executive<br>2. Prioritisation of health over finance<br>2.1 Health service as a health-promoting setting<br>2.2 'Right' thing to do<br>2.3 Low risk of loss of vending revenue<br>2.4 Leading by example<br>3. Dietitian working closely with the vending supplier<br>3.1 Helping supplier research and find 'green' food options<br>3.2 Educating the supplier's staff on nutrition<br>4. Existence of state government guidelines<br>4.1 Platform for policy change<br>4.2 Supports the credibility of the policy<br>4.3 Used as a resource in implementation<br><br>Three themes related to factors likely to directly impact on sales of foods and beverages and revenue to health service<br>1. Creation of new vendor contract<br>1.1 Increased commission rate (of hospital) lowered financial risk<br>2. Healthier options in vending<br>2.1 Difficulty in obtaining 'green' options<br>2.2 Contract required supplier to source and stock 'green' products<br>2.3 Industry movement towards reformulation created more 'amber' food options<br>2.4 Future opportunities in refrigerated vending<br>3. Optimising machine placement<br>3.1 Replacing low earning machines and moving machines to high-traffic areas

Study, publications Location	Summary of key findings (Theme and sub-theme names outlines in publications were retained where possible)
**Healthy Choices in Alfred Health** (2019[5] journal publication)<br><br>Boelsen-Robinson et al. (2019)[5];<br>Victorian Health Promotion Foundation (2017)[6];<br>Boelsen-Robinson et al. (2016)[7];<br>Boelsen-Robinson (2019)[4]<br><br>Victoria, Australia	Four main themes:<br>1. Resources and support<br>1.1 Increased time and effort for service staff<br>1.2 Dietitians accessible<br>1.3 Senior executive as champion<br>1.4 Appointment of health promotion manager<br>1.5 Increased time for supplier<br>2. Communication<br>2.1 Engagement with food outlet owner<br>2.2 Public acknowledgment of success<br>2.3 Communication with customers<br>3. Tension and balance<br>3.1 Initial scepticism and concern from food retail staff<br>3.2 Negative customer reactions<br>3.3 Disruption of standard chef practices<br>3.4 Balancing priorities of health and financial viability<br>3.5 Change in ownership of retail outlet<br>4. Passion<br>4.1 Health promotion manager dedication<br>4.2 Opportunity to contribute to public health<br>4.3 Healthy food as a competitive advantage<br>4.4 Provision of customer service

Study, publications Location	Summary of key findings (Theme and sub-theme names outlines in publications were retained where possible)
**Victorian Healthy Choices and Healthy Catering case studies**<br><br>Chang et al. (2016)[8]<br><br>Melbourne Inner East Catchment, Victoria, Australia	1. Key barriers to implementation<br>1.1 Maintaining consistent supply and variety of healthy options in vending machines<br>1.2 Perception that customers do not want healthier foods<br>1.3 Loss of interest in implementation over time<br>1.4 Classification of foods using guidelines by retailers may be too complex<br><br>2. Key facilitators to implementation<br>2.1 Leadership at every level and continuous involvement with stakeholders<br>2.2 Community involvement creating ownership over initiatives and enabling their sustainability<br>2.3 Clear, effective communication of guidelines and its content to retailers, caterers and staff<br>2.4 Support from nutrition professionals enabling clarity and understanding of guidelines<br>2.5 Stable profits as unhealthy purchases are replaced with healthier options<br>2.6 Piloting short implementation periods allowing gradual introduction of guidelines
**Healthy Choices in Aboriginal ACCHOs**<br><br>MacDonald et al. (2016)[9]<br><br>Victoria, Australia	1. Key barriers to implementation<br>1.1 Lack of policy endorsement by management (competing management agendas)<br>1.2 Limited Aboriginal nutrition workforce (multiple responsibilities of project champions in workplaces)<br>1.3 Staff turnover within workforce responsible for implementation<br>1.4 Under-resourced support and administration departments<br><br>2. Key facilitators to implementation<br>2.1 Project champions enabling, supporting and advocating for change<br>2.2 Support and backing from management<br>2.3 Tools and resources supporting policy implementation

Study, publications Location	Summary of key findings (Theme and sub-theme names outlines in publications were retained where possible)
**Victorian policies in sport and recreation facilities**<br><br>Riesenberg et al. (2020)[10]<br><br>Victoria, Australia	1. Key barriers to implementation<br>1.1 Inadequate support from key stakeholders (local government members, leadership teams)<br>1.2 Inadequate funding (especially those facilities in lower density and rural areas)<br>1.3 Inadequate control over facilities<br>1.4 Inadequate time<br>1.5 Inability to source appropriate healthy alternative<br>1.6 Disagreeable suppliers<br>2. Key enablers to implementation<br>2.1 Support from key stakeholders (main enabler)<br>2.2 Appropriate funding<br>2.3 Adequate control over facilities<br>2.4 Adequate time<br>2.5 Ability to source appropriate healthy alternatives<br>2.6 Agreeable suppliers
**A Better Choice evaluation**<br><br>Miller et al. (2015)[11]; Queensland Health (2010)[12]<br><br>Queensland, Australia	1. Key barriers to implementation<br>1.1 Perceived customer dissatisfaction with limitation of 'red' category foods and drinks<br>1.2 Difficulty accessing suitable 'green' category products<br>1.3 Perceived lack of demand for healthy foods and drinks<br>1.4 Concern over loss of profit<br>1.5 Lack of management support<br>2. Key facilitators to implementation<br>2.1 Support from leadership, local food service staff, nutritionist/dietitian and staff members<br>2.2 Access to strategy, catering documents, and promotional materials<br>2.3 Mandating policy by Queensland Health<br>2.4 Communication strategies to increase staff awareness<br>2.5 Regular compliance audits

Study, publications Location	Summary of key findings (Theme and sub-theme names outlines in publications were retained where possible)
**Queensland children's hospital study**<br><br>Walker et al. (2020)[13]<br><br>Brisbane, Queensland, Australia	1. Key concerns associated with implementation<br>1.1 Inadequate notice about upcoming changes given to customers<br>2. Key facilitators to implementation<br>2.1 Tailored implementation guide<br>2.2 Communication strategy (discussions and negotiations with implementation team)<br>2.3 Adequate time allocation for required tasks
**Healthy Options WA Department of Health, evaluation**<br><br>Western Australia Department of Health (2020)[14]<br><br>Western Australia, Australia	3. Key barriers to implementation<br>3.1 Concerns regarding potential profit loss<br>3.2 Difficulty in finding products to align with the Policy<br>3.3 Confusion about and difficulty understanding the Policy<br>4. Key enablers to implementation<br>4.1 Support from dietitian/health promotion staff<br>4.2 The Healthy Option Implementation Guide<br>4.3 Support from hospital management<br>5. Awareness of existing resources to support policy implementation among food outlet managers and staff was low<br>6. Compliance with the Policy requirements in business and professional catering and fundraising requires future focus

Study, publications Location	Summary of key findings (Theme and sub-theme names outlines in publications were retained where possible)
**Healthy Options WA in East Metropolitan Health Service**<br><br>Law et al. (2021)[15]<br><br>Perth, Western Australia, Australia	Three main themes:<br>1. Food retailers had come to accept their role in implementing the policy<br>1.1 It's fair when everyone has to do it (fairness seen as implementation of policy by all retailers at once as communicated by the executive leadership; unfairness seen as no requirement for the food outlets nearby or food trucks on site to also comply with the policy)<br>1.2 Willing to make hard changes (including noticeable and behind-the-scenes changes)<br>1.3 Duplication of efforts is frustrating (sourcing and classifying products against the policy criteria requiring extra time repeated by all retailers indicating need for centralised electronic list of available products)<br>2. The policy made it difficult for food retailers to operate successfully<br>2.1 Pressure of conflicting demands (complying with policy criteria while running a financially viable business and meeting customer demands)<br>2.2 At the front lines with customers (customer dissatisfaction and at times abuse, especially from hospital staff, resulting in training staff to respond to customers positively)<br>2.3 Creates extra work for no benefits (duties going beyond the scope of retailers' roles)<br>3. Food retailers needed help and support to implement the policy<br>3.1 Confusion (policy nutrient criteria) and misinformation (from suppliers about their products)<br>3.2 Timely support from a trusted source (mainly Public Health Dietitian) is essential<br>3.3 Sourcing affordable (and profitable for retailers), acceptable 'green' products is hard (especially snacks and long shelf-life products)<br>3.4 Some rules seem wrong (or inconsistent) or unachievable

Study, publications Location	Summary of key findings (Theme and sub-theme names outlines in publications were retained where possible)
**South Australian Health facilities evaluation**<br><br>Government of South Australia (2012)[16]<br><br>South Australia, Australia	1. Key barriers to implementation<br>1.1 Difficulties with external contractors<br>1.2 Difficulties changing workplace culture<br>1.3 Staff attitudes<br>2. Key facilitators to implementation<br>2.1 Regular communication to staff<br>2.2 Positive staff attitudes<br>2.3 Engagement with stakeholders<br>2.4 Use of policy resources
**Army DFACs intervention**<br><br>Armstrong et al. (2020)[17]<br><br>Fort Bragg, North Carolina, USA	1. Barriers based on food service employees' experience<br>1.1 Nutrition knowledge deficits<br>1.2 Inadequate culinary training<br>1.3 Poor management practices<br>1.4 Low staff morale
**Hubert Building Cafeteria Guidelines**<br><br>Bayne et al. (2012)[18]<br><br>Washington, District of Columbia, USA	1. Key challenges to implementation<br>1.1 Meeting the sodium guidelines (limited availability of low-sodium products on the market)<br>1.2 Sourcing speciality products (healthier products available only through bulk-buying and not stocked by distributors)<br>1.3 Access to local and organic produce<br>1.4 Higher cost of healthier products<br>1.5 Customer acceptance<br>2. Key facilitator to implementation<br>2.1 Leadership and commitment at all levels<br>2.2 Availability of dietitians and dedicated implementation staff<br>2.3 Customer education, awareness and support for changes<br>2.4 Large size of the workplace

**Study, publications Location**	**Summary of key findings** (Theme and sub-theme names outlines in publications were retained where possible)
**Boston Sodium Reduction Initiative**<br><br>Brooks et al. (2017)[19]<br><br>Boston, Massachusetts, USA	1. Implementation of sodium-reduction intervention in community settings is feasible, although more challenging in vending machines<br>2. Key barrier to implementation<br>2.1 Lack of knowledge who had control of stocking vending machines in own institutions<br>2.2 Little control over vending machine stocking practices<br>3. Key facilitators to implementation<br>3.1 Leveraging purchasing power in contract negotiations with vendors<br>3.2 Awarding contracts to and working with local suppliers<br>3.3 Tailored technical assistance
**Vidant Healthy Food Environment Policy**<br><br>Gaskins et al. (2013)[20]<br><br>Greenville, North Carolina, USA	1. Key barriers to implementation<br>1.1 Lack of food products that comply with guidelines<br>1.2 Accurate ingredient measurements and consistent portion control<br>1.3 Keeping customer interest<br>1.4 Financial management shared by food service operator and a volunteer organisation and distributing café's profits to hospital for needed programmes, equipment and services<br>2. Key facilitators to implementation<br>2.1 Opportunities to gradually improve food environments<br>2.2 Communication and consultation with key stakeholders<br>2.3 Dedicated champion guiding implementation

**Study, publications Location**	**Summary of key findings** (Theme and sub-theme names outlines in publications were retained where possible)
**Healthy Vending Policies in US cities**<br><br>Green et al. (2020)[21]<br><br>Four cities in USA (2 large, 2 smaller)	1. Key barriers to implementation<br>1.1 Conducting regular compliance checks of vending machines<br>1.2 Inability to obtain sales data<br>1.3 Concerns about profit loss<br>2. Key facilitators to implementation<br>2.1 Having a champion and support from leadership<br>2.2 Internal and external partnership<br>2.3 Clear communication
**US Hospital and Federal Worksites Guidelines**<br><br>Jilcott Pitts et al. (2016)[22]<br><br>USA (various locations)	1. Key barriers to implementation<br>1.1 Customer complaints<br>1.2 Shortage of foods and beverages from vendors that met the required guidelines<br>2. Key facilitators to implementation<br>2.1 Leadership support<br>2.2 Corporate commitment to healthy changes<br>2.3 Collaborative vendor partnership<br>2.4 Open communication with food service workers and vendors<br>2.5 Registered dietitians to provide health education, coordinate and conduct programme activities, and manage monitoring and implementation

Study, publications Location	Summary of key findings (Theme and sub-theme names outlines in publications were retained where possible)
**US Hospital food service guidelines**<br><br>Jilcott Pitts et al. (2018)[23]<br><br>USA	1. Findings related to demand for and sales of healthy food and beverages<br>1.1 Food service operators worried higher prices of healthy foods and highly restrictive healthy guidelines will decrease customer numbers and profits<br>1.2 Food service operators found discontinuing sales of comfort and familiar foods challenging<br>1.3 Stakeholders employed various strategies to offset potential loss of customers and sales (e.g. taste tasting, discounts for healthy foods, communicating changes in advance)<br>2. Findings related to production and supply of healthy food and beverages<br>2.1 Limited healthy food options from suppliers led to more costly preparation of healthy foods on-site (staff time, recipe development, higher cost of healthy ingredients)<br>2.2 Online food service staff training delivered by executive chef facilitated implementation<br>2.3 Successful implementation requires upfront capital investment
**NYC Healthy Hospital Food Initiative**<br><br>Lederer et al. (2014)[24]<br><br>New York City, New York, USA	1. Key barriers to implementation<br>1.1 Perceived lack of demand for/education about healthy foods<br>1.2 Perceived lack of customers' support/satisfaction with healthier options<br>1.3 Logistical and cost barriers associated with purchasing lower sodium items<br>1.4 Layout of food outlets<br>1.5 Competition from outside retailers<br>1.6 Lack of upper management support
**'Red Apple' Hospitals Project**<br><br>Neffa (2011)[25]<br><br>North Carolina, USA	1. Key facilitators to implementation<br>1.1 Receiving feedback about customer adoption/acceptance of the project<br>1.2 Support from an external partner organisation (online tools, flexible guidelines, hospital networks, encouragement)<br>1.3 Support from the hospital's senior managers (moral and financial support)<br>1.4 Support form a diverse, committed team of implementers

Study, publications Location	Summary of key findings (Theme and sub-theme names outlines in publications were retained where possible)
**San Antonio Sodium Reduction Initiative**<br><br>Sosa et al. (2019)[26];<br>Ullevig et al. (2019)[27]<br><br>Bexar County, Texas, USA	1. Key barriers to implementation<br>1.1 Concerns with customer dissatisfaction with low sodium options<br>1.2 Higher cost and low availability of lower sodium ingredient alternatives<br>1.3 Time constraints<br>2. Key facilitators to implementation<br>2.1 Expertise of the Sodium Reduction Initiative team (technical support)<br>2.2 Ability to make iterative and gradual changes to reduce sodium in meals<br>2.3 Suppliers with large selection of healthier options<br>2.4 Organisational culture of wellness (existing concurrent health initiatives and buy-in from stakeholders)
**Washington State's Healthy Nutrition Guidelines Executive Order**<br><br>Otten et al. (2014)[28];<br>Otten et al. (2015)[29];<br>Podrabsky et al. (2016)[30];<br>Podrabsky et al. (2018)[31]<br><br>Washington State, USA	1. Key barriers to implementation<br>1.1 Lack or insufficient communication<br>1.2 Lack or insufficient support and resources<br>1.3 Competing priorities<br>1.4 Ambiguity about specific roles and duties<br>1.5 Concerns over implementation costs and loss of profits<br>1.6 Lack of clarity around the guideline's content and purpose<br>1.7 Voluntary nature of guidelines<br>2. Key facilitators to implementation<br>2.1 Communication with agency representative<br>2.2 Internal and external support<br>2.3 Positive response from customers<br>2.4 Personal motivation of cafeteria operators
**City of Hamilton Policy**<br><br>Atkey et al. (2017)[32]<br><br>City of Hamilton, Ontario, Canada	1. Piloting of policies allows to identify barriers to implementation and strategize further implementation<br>2. Implementation progresses faster for catered staff functions and slower for vending machines and concession stands<br>3. Support for policy implementation as key facilitator (dedicated personnel, resources, interest from upper-management)<br>4. Different level of implementation across workplace departments as key barrier

Study, publications Location	Summary of key findings (Theme and sub-theme names outlines in publications were retained where possible)
**Healthy Foods in Champlain Hospitals**<br><br>Dojeiji et al. (2017)[33]<br><br>Champlain region of Eastern Ontario, Canada	1. Key barriers to implementation<br>1.1 Limited availability of healthier vending products<br>1.2 Minimum ordering requirements<br>1.3 Limited ability to make changes to established menus in franchises (existing contractual agreements)<br>1.4 Concerns about potential profit losses<br>2. Key facilitators to implementation<br>2.1 Conducting staff surveys and taste testing sessions to identify desirable healthier options<br>2.2 Combining orders across departments to generate required ordering volumes<br>2.3 Shared accountability and supportive approaches to reduce barriers<br>2.4 Financial, leadership and dietetic support
**Three Canadian provinces study**<br><br>Kirk et al. (2021)[34]<br><br>British Columbia, Alberta and Nova Scotia; Canada	1. Barriers to implementation:<br>1.1 Unhealthy food culture within community<br>1.2 Competition from other food providers outside the facilities<br>1.3 Issues within food service contracts (long-term contracts, marketing obligations)<br>2. Facilitators to implementations:<br>2.1 Provincial or municipal expectations of guideline implementation<br>2.2 Clear communication to staff around guideline directives<br>2.3 Presence of a champion within the community or facility who supported guideline implementation<br>2.4 Increasing demand from customers for healthier food choices<br>2.5 Availability of data demonstrating an increase in sales of healthier foods
**Canadian Healthy Eating in Recreation and Sport Settings**<br><br>McIsaac et al. (2018)[35]<br><br>Nova Scotia, Canada	1. Key barriers to implementation:<br>1.1 Cultural norms associated with unhealthy food in recreation and sport settings and the broader environment<br>1.2 Persisting notion of consumers' personal choice and responsibility of healthy eating<br>1.3 Perceived negative financial implications of healthy food provision

**Study, publications Location**	**Summary of key findings** (Theme and sub-theme names outlines in publications were retained where possible)
**Healthy Food and Beverage Sales Phase I pilot study**<br><br>Naylor et al. (2010)[36]<br><br>British Columbia, Canada	1. Barriers to implementation<br>1.1 Lack of a dedicated person to facilitate implementation<br>1.2 Gaining buy-in from decision makers, staff, industry and public<br>1.3 Lack of policy guidelines<br>1.4 Time constrains<br>2. Facilitators to implementation:<br>2.1 Hiring a contractor with nutrition background<br>2.2 Provincial policy guidelines<br>2.3 Evaluation process<br>2.4 Communication with stakeholders<br>2.5 Funding (money and resources)<br>2.6 Technical support from Healthy Food and Beverage Sales initiative
**Healthy Food and Beverage Sales Phase II study**<br><br>Vander Wekken & Naylor (2010)[37]<br><br>British Columbia (BC), Canada	1. Key implementation challenges:<br>1.1 Lack of buy-in from facility staff, patrons, local decision makers, vendors and concessionaires<br>1.2 Revenue loss<br>1.3 Limited resources including staff time and energy, money, information<br>1.4 Finding suitable products<br>1.5 Competition in food environments<br>1.6 Lack of supportive policy<br>2. Key implementation facilitators:<br>2.1 Supportive policy and buy-in from local decision makers<br>2.2 Buy-in and support from staff, management, patrons, community groups and the snack and beverage industry (including vendors)<br>2.3 Resources including time, money, staff time and resources provided through the HFBS initiative<br>2.4 Information sharing between communities<br>2.5 The evaluation process<br>3. The main finding of the process evaluation was that "change takes time and resources" and is best accomplished with "buy-in from everyone"

Study, publications Location	Summary of key findings (Theme and sub-theme names outlines in publications were retained where possible)
**Healthy Food and Beverage Sales controlled study**<br><br>Naylor et al. (2015)[38]<br><br>British Columbia, Canada	1. Barriers to implementation<br>1.1 Concern of revenue loss and associated costs<br>1.2 Lack of viable healthy choice products<br>1.3 Lack of resources to support making change<br>1.4 Lack of local decision-maker buy-in<br>1.5 Limited staff resources<br>1.6 Patron demand (for unhealthy foods)<br>1.7 Legislation with bottled water (council-decision not to serve bottled water that is a healthy alternative in the policy)<br>2. Facilitators to implementation<br>2.1 Stakeholder buy-in<br>2.2 Availability of resources<br>2.3 Sharing information<br>2.4 Evaluation process<br>2.5 Having policy in place<br>2.6 Being a part of a bigger movement

Study, publications Location	Summary of key findings (Theme and sub-theme names outlines in publications were retained where possible)
**Healthy Food and Beverage Sales - Industry Perspectives**<br><br>Vander Wekken et al. (2012)[39]<br><br>British Columbia (BC), Canada	Key themes:<br>1. Perceived limitations of the policy<br>1.1 Insufficient input from the industry during development<br>1.2 Guidelines are overwhelming<br>2. Stocking healthy products<br>2.1 Limited variety<br>2.2 Limited suppliers<br>2.3 Patron complains<br>2.4 Additional waste and service costs<br>3. Competition in food sales environments<br>3.1 Competition within a facility<br>3.2 Competition outside a facility<br>4. Negative impact on profits and business survival<br>4.1 Cost and risk of investing in healthy products<br>4.2 Fewer customers buy the healthy-choice option<br>4.3 Those customers who support healthy eating don’t buy vending products<br>4.4 Downsizing and concerns with business survival<br>5. Using policy as a standardised definition of healthy and less healthy products<br>6. Support services (dietitians and list of compliant packaged foods and drinks)<br>7. Future trends and opportunities<br>7.1 Ethical consumer interest (sustainability, fair-trade)<br>7.2 Healthy lifestyles<br>7.3 New markets<br>7.4 Slow generational transition to accepting healthier vending options<br>7.5 Changing perceptions of vending industry to healthier image

Study, publications Location	Summary of key findings (Theme and sub-theme names outlines in publications were retained where possible)
**Healthier Recreation Concession Pilot Project**<br><br>Neil & Haile (2016)[40]<br><br>Oxford County, Ontario, Canada	1. Recreation centres play an important preventative role in public health<br>2. Early support from Public Health Dietitian helped eased implementation<br>3. Patrons had positive feedback about the changes<br>4. Implementation takes time and requires an array of promotional strategies<br>5. Operator noted several difficulties with implementation (short shelf-life of products, higher wastage, patrons requesting the unhealthier items or purchasing them elsewhere)
**South Korean Sodium reduction pilot project**<br><br>Lee & Park (2016)[41]<br><br>South Korea	1. Those participants that received education on the significance of sodium reduction, cooking methods and using alternative seasonings for sodium-reduced meals, placed more importance on provision of sodium-reduced meals<br>2. Key barriers to implementation<br>2.1 Use of processed foods that are high in sodium<br>2.2 Limitation of sodium-reduced cooking methods<br>2.3 Limited number of sodium-reduced menu items<br>2.4 Difficulties in serving sodium-reduced meals due to increased cost and lack of personnel

Study, publications Location	Summary of key findings (Theme and sub-theme names outlines in publications were retained where possible)
**South Korean reduced-sodium meals national programme**<br><br>Park & Lee (2016)[42]<br><br>South Korea	Four main themes<br>1. Key stakeholders' psychological characteristics (facilitators)<br>1.1 Positive perceptions, intentions and preferences among customers<br>1.2 Positive attitudes towards low-sodium diets among food-service personnel<br>2. Skills and techniques relevant to serving reduced-sodium meals (barriers)<br>2.1 Restricted resources for menu planning<br>2.2 Limited cooking skills for reduced-sodium meals<br>2.3 Lack of skills for measuring sodium content quickly<br>3. Support from headquarters and government (barriers)<br>3.1 Lack of variety in low-sodium menus<br>3.2 Lack of a nutrient database for estimating sodium content easily and quickly<br>4. Social, physical and economic environments<br>4.1 Social consensus on the health benefits of lowering dietary sodium intake (facilitator)<br>4.2 Lack of staff (barrier)<br>4.3 Limited space in kitchens and halls (barrier)<br>4.4 Pressure on sales (barrier)<br>4.5 Tight profit margin and tight budget (barrier)
**Dutch environmental nutrition programme intervention**<br><br>Steenhuis et al. (2004)[43]<br><br>Netherlands	1. Educational programmes directed at employees need to be captivating and intensively promoted as part of implementation strategy<br>2. Addition of healthier options to the current range without decreasing unhealthy options is not enough to shift consumer buying habits<br>3. Healthier alternatives are hard to find and source from distributors

Study, publications Location	Summary of key findings (Theme and sub-theme names outlines in publications were retained where possible)
**Dutch portion size and pricing intervention**<br><br>Vermeer et al. (2012)[44]<br><br>Netherlands	1. Offering additional smaller portions is not complex, time-consuming or risky for business<br>2. Demand for smaller portion sizes varied by worksites<br>3. Managers input and supervision facilitated food service staff to follow intervention's protocol
**Scottish Healthcare Retail Standard**<br><br>Stead et al. (2020)[45];<br>Shipton (2019)[46]<br><br>Scotland	1. Managers were largely not supportive of the standards, with more positive attitudes after implementation<br>2. Implementation was successful across different retail outlets<br>3. Key challenges to implementation:<br>3.1 Limited resources and lack of knowledge (identified by smaller independent food outlet managers)<br>3.2 Limited availability and high cost of healthier items<br>3.3 Little or no awareness raising of the standards with customers (identified as a missed opportunity) resulting in customer complains<br>3.4 Decrease in sales post-implementation (gradual increase over time although not to pre-implementation levels)<br>4. Key facilitators to implementation:<br>3.1 Relatively long lead-in time for implementation<br>3.2 Building on existing business relationships<br>3.3 Support from upper management and implementation team<br>3.4 Product reformulation by suppliers
**Welsh Hospital Vending**<br><br>Welsh Assembly Government (2009)[47]<br><br>Wales	1. Supply-side stakeholders were generally supporting of healthy vending guidance, but would prefer a phased approach and more consultation at guidance development stage<br>2. Implementation had a negative impact on sales and customer satisfaction<br>3. Supply-side stakeholders want to offer healthy foods and drinks, but currently there is no suitable and affordable alternatives on the market and the design of vending machines does not fit the packaging dimensions of healthier products<br>4. Welsh Assembly Government to continue communication and engagement with key stakeholders and customer education

Study, publications Location	Summary of key findings (Theme and sub-theme names outlines in publications were retained where possible)
**East London Food for Life initiative**<br><br>Gray et al. (2017)[48]<br><br>East London, West Yorkshire, and South Warwickshire, England	1. Developing and implementing nutrition standards seen as common goal and process<br>2. Contractual agreements can be either a barrier or enabler to changes in workplaces<br>3. Changes in food quality improved customer satisfaction<br>4. Health facilities viewed as role models of healthy eating<br>5. More focus should be placed on foods available out of normal cafeteria operating hours
**"Healthful & Tasty: Sure" Sodium Reduction Trial**<br><br>Beer-Borst et al. (2018)[49] (Protocol);<br>Beer-Borst et al. (2019)[50];<br>Beer-Borst et al. (2020)[51]<br><br>German-speaking part of Switzerland	1. Sodium reduction depends on strong upper management/employer support<br>2. Environmental factors need to be taken into consideration when implementing healthier eating guidelines<br>3. Barriers to implementation included lack of skilled personnel, individual cooking and taste preferences, presence of convenience foods, worries that taste-detectable reduction in salt content may reduce sales
**Danish '6 a day' Worksite Canteen study**<br><br>Lassen et al. (2004)[52]<br><br>Denmark	1. Key facilitators to implementation<br>1.1 Leadership support<br>1.2 Getting all staff involved in implementation process<br>1.3 Use of various strategies to increase fruit and vegetable content of meals

Study, publications Location	Summary of key findings (Theme and sub-theme names outlines in publications were retained where possible)
**New Zealand Heartbeat Programme**<br><br>Young et al. (2004)[53]<br><br>New Zealand	1. Facilitators to implementing catering programme were catering toolkits, recipe ideas, cooking demonstrations, newsletters<br>2. Implementation resources should be regularly updated, visual and user-friendly<br>3. Caterers increased their nutrition knowledge through the catering programme

References

1. Blake MR, Boelsen-Robinson T, Hanna L, Ryan A, Peeters A. Implementing a healthy food retail policy: a mixed-methods investigation of change in stakeholders' perspectives over time. *Public Health Nutr*. 2020;24(9):2669-2680.

2. Boelsen-Robinson T, Backholer K, Corben K, Blake MR, Palermo C, Peeters A. The effect of a change to healthy vending in a major Australian health service on sales of healthy and unhealthy food and beverages. *Appetite*. 2017;114:73-81.

3. Peeters A, Corben K, Boelsen-Robinson T. Spotlight 3.2 Integrating healthy food provision and economic viability in a large metropolitan health service, Australia. In: *Global Nutrition Report. Nourishing the SDGs.* Bristol, UK: Development Initiatives; 2017:59.

4. Boelsen-Robinson T. Healthy food policy implementation for retailers in health-promoting settings: building the evidence. 2019.

5. Boelsen-Robinson T, Blake MR, Backholer K, Hettiarachchi J, Palermo C, Peeters A. Implementing healthy food policies in health services: A qualitative study. *Nutr Diet*. 2019;76(3):336-343.

6. Victorian Health Promotion Foundation. *Introduction of Healthy Choices at Alfred Health Food Outlets. Evaluation Summary*. Victoria, Australia: Victorian Government; 2017. https://www.vichealth.vic.gov.au/-/media/ResourceCentre/PublicationsandResources/healthy-eating/Healthy-Choice/Introduction-of-Healthy-Choices-at-Alfred-Health-food-outlets.pdf. Accessed July 9, 2020.

7. Boelsen-Robinson T, Blake M, Hettiarachchi J, Palermo C, Backholer K, Peeters A. Characterising factors associated with the implementation of a healthy food policy-an analysis of a major Australian health service. *Obes Rev*. 2016;17(Suppl. 2):157.

8. Chang J, Due D, Khoo A, Paciepnik J. A case for change: Gathering the evidence for healthy food and drink initiatives. 2016. https://iepcp.org.au/wp-content/uploads/2017/01/A-Case-for-Change-Report.pdf.

9. MacDonald C, Genat B, Thorpe S, Browne J. Establishing health-promoting workplaces in Aboriginal community organisations: healthy eating policies. *Aust J Prim Health*. 2016;22(3):239-243.

10. Riesenberg D, Blake MR, Boelsen-Robinson T, Peeters A, Cameron AJ. Policies influencing the provision of healthy food and drinks in local government-owned sport and recreation facilities in Victoria, Australia. *Aust NZ J Public Health*. 2020;44(3):240-244.

11. Miller J, Lee A, Obersky N, Edwards R. Implementation of A Better Choice Healthy Food and Drink Supply Strategy for staff and visitors in government-owned health facilities in Queensland, Australia. *Public Health Nutr*. 2015;18(9):1602-1609.

12. Queensland Health. *A Better Choice Healthy Food and Drink Supply Strategy for Queensland Health Facilities: Evaluation Report*. Brisbane, Australia: Queensland Health; 2010:45. www.health.qld.gov.au. Accessed April 8, 2021.

13. Walker JL, Littlewood R, Rogany A, Capra S. Implementation of the "Healthier Drinks at Healthcare Facilities" strategy at a major tertiary children's hospital in Brisbane, Australia. *Aust NZ J Public Health*. 2020;44(4):295-300.

14. Western Australia Department of Health. *Healthy Options WA: Food and Nutrition Policy for WA Health Services and Facilities 2018–19 Statewide Audit of Policy Implementation*. Perth, Australia: Government of Western Australia; 2020. https://ww2.health.wa.gov.au/~/media/Files/Corporate/Reports-and-publications/Audit-of-food-and-drink/2018-2019-Healthy-Options-WA-Policy-Statewide-Audit-of-Policy-Implementation-Report.pdf.

15. Law KK, Pulker CE, Healy JD, Pollard CM. "Just so you know, it has been hard": food retailers' perspectives of implementing a food and nutrition policy in public healthcare settings. *Nutrients*. 2021;13(6):2053.

16. Government of South Australia. *Healthy Food and Drink Choices for Staff and Visitors in SA Health Facilities Policy Evaluation: Summary Report of Findings*. South Australia, Australia; 2012. Available at: https://www.sahealth.sa.gov.au/wps/wcm/connect/public+content/sa+health+internet/public+health/preventative+health+and+wellbeing/healthy+food+and+drink+choices+in+sa+health+facilities.

17. Armstrong NJ, Serrano EE, Cole RE, Bukhari AS, Jayne JM. Barriers to nutrition interventions in Army Dining Facilities: A qualitative study. *Mil Med*. 2020:1-6.

18. Bayne AI, Hair E, Harris Brewer K, Garg A. *The HHS Hubert H. Humphrey Building Cafeteria Experience: Incorporation of the Dietary Guidelines for Americans, 2010 into Federal Food Service Guidelines. Prepared by NORC at the University of Chicago.* Washington, DC: U.S: Department of Health and Human Services, Office of the Assistant Secretary for Planning and Evaluation.; 2012.

19. Brooks CJ, Barrett J, Daly J, et al. A community-level sodium reduction intervention, Boston, 2013–2015. *Am J Public Health*. 2017;107(12):1951-1957.

20. Gaskins S, Kolasa K, Simmons RA, Njuguna N, White A. Creating Vidant Health's healthy food environment. *Top Clin Nutr*. 2013;28(2):189-199.

21. Green S, Glanz K, Bromberg J. Facilitators and barriers to developing, implementing, and evaluating healthy vending policies in four cities. *Health Promot Pract*. 2020:1-6.

22. Jilcott Pitts SB, Graham J, Mojica A, et al. Implementing healthier foodservice guidelines in hospital and federal worksite cafeterias: barriers, facilitators and keys to success. *J Hum Nutr Diet*. 2016;29(6):677-686.

23. Jilcott Pitts S, Schwartz B, Graham J, et al. Best practices for financial sustainability of healthy food service guidelines in hospital cafeterias. *Prev Chronic Dis*. 2018;15(E58):170477.

24. Lederer A, Toner C, Krepp EM, Curtis CJ. Understanding hospital cafeterias: results from cafeteria manager interviews. *J Public Health Manag Pract*. 2014;20(1):S50-S53.

25. Neffa D. The "Red Apple" Healthy Food Environments in Hospitals Project: A qualitative analysis of the implementation process. 2011.

26. Sosa ET, Ullevig SL, Spitsen E, Heine A, Perez C, Shields K. The San Antonio Sodium Reduction Initiative: lessons learned and recommendations. *Am J Health Educ*. 2019;50(3):200-205.

27. Ullevig SL, Spitsen E, Heine A, Shields K, Sosa ET. Impact of a Local Sodium Reduction Initiative at hospital cafeterias. *J Acad Nutr Diet*. 2019;119(Suppl 1):A80.

28. Otten J, Bachaus J, Hulbrock E. *Evaluating Washington State's Healthy Nutrition Guidelines: Report from a Baseline Evaluation of Executive Order 13-06*. Washington State, USA: Washington State Department of Health; 2014:33. Available at: https://doh.wa.gov/community-and-environment/worksite-wellness/healthy-nutrition-guidelines.

29. Otten J, Podrabsky M, Althauser A, Diedrich S, Johnston S. *Implementation of Washington State's Healthy Nutrition Guidelines Under Executive Order 13-06: Mid-Term Evaluation*. Washington State, USA: Washington State Department of Health; 2015. Available at: https://doh.wa.gov/community-and-environment/worksite-wellness/healthy-nutrition-guidelines.

30. Podrabsky M, Rice L, Ritter G, Otten J. *Implementation of Washington State's Healthy Nutrition Guidelines Under Executive Order 13-06: 2016 Evaluation*. Washington State, USA: Washington State Department of Health; 2016. Available at: https://doh.wa.gov/community-and-environment/worksite-wellness/healthy-nutrition-guidelines.

31. Podrabsky M, Walkinshaw LP, Bradford V, Beck L, Otten Jennifer J. *Implementation of Washington State's Healthy Nutrition Guidelines Under Executive Order 13-06: 2018 Evaluation*. Washington State, USA: Washington State Department of Health; 2018. Available at: https://doh.wa.gov/community-and-environment/worksite-wellness/healthy-nutrition-guidelines.

32. Atkey K, Elliott-Moyer P, Freimanis M, Raine KD. Stories of policy change: City of Hamilton's healthy food and beverage policy. *Can J Public Health*. 2017;108(5-6):e625-e629.

33. Dojeiji L, Taylor A, Boland C, Brennan C, Penney R. Retail food reform: How to effectively bridge what we say and what we do in our hospital settings. *Healthc Manage Forum*. 2017;30(2):101-106.

34. Kirk SFL, Olstad DL, McIsaac JLD, et al. Appetite for change? Facilitators and barriers to nutrition guideline implementation in Canadian recreational facilities. *Health Promot Internation*. 2021:1-11.

35. McIsaac JLD, Jarvis SL, Spencer R, Kirk SFL. "A tough sell": findings from a qualitative analysis on the provision of healthy foods in recreation and sports settings. *Health Promot Chronic Dis Prev Can*. 2018;38(1):18-22.

36. Naylor PJ, Vander Wekken S, Trill D, Kirbyson A. Facilitating healthier food environments in public recreation facilities: results of a pilot project in British Columbia, Canada. *J Park Recreat Admi*. 2010;28(4):37-58.

37. Vander Wekken S, Naylor PJ. *Healthy Food and Beverage Sales In Recreation Facilities and Local Government Buildings. Phase II Facilities Evaluation Summary Report*. British Columbia, Canada: University of Victoria; 2010. https://stayactiveeathealthy.ca/wp-content/uploads/formidable/6/HFBS-Phase-II-Evaluation-Summary-Report_Revised-April-20-2010.pdf.

38. Naylor PJ, Olstad DL, Therrien S. An intervention to enhance the food environment in public recreation and sport settings: a natural experiment in British Columbia, Canada. *Child Obes*. 2015;11(4):364-374.

39. Vander Wekken S, Sørensen S, Meldrum J, Naylor PJ. Exploring industry perspectives on implementation of a provincial policy for food and beverage sales in publicly funded recreation facilities. *Health Policy*. 2012;104(3):279-287.

40. Neil K, Haile R. *A Healthier Recreation Concession Pilot Project: Evaluation Report*. Woodstock, Ontario, Canada: Oxford County Public Health; 2016. http://www.oxfordcounty.ca/Portals/15/Documents/BB%20A%20Healthier%20Recreation%20Concession%20Pilot%20Evaluation%20July%202016.pdf.

41. Lee J, Park S. Management of sodium-reduced meals at worksite cafeterias: perceptions, practices, barriers, and needs among food service personnel. *Osong Public Health Res Perspect*. 2016;7(2):119-126.

42. Park S, Lee J. "When operating a cafeteria, sales come before nutrition" - finding barriers and facilitators to serving reduced-sodium meals in worksite cafeterias. *Public Health Nutr*. 2016;19(8):1506-1516.

43. Steenhuis I, van Assema P, Reubsaet A, Kok G. Process evaluation of two environmental nutrition programmes and an educational nutrition programme conducted at supermarkets and worksite cafeterias in the Netherlands. *J Hum Nutr Diet*. 2004;17(2):107-115.

44. Vermeer WM, Leeuwis FH, Koprulu S, Zouitni O, Seidell JC, Steenhuis IHM. The process evaluation of two interventions aimed at portion size in worksite cafeterias. *J Hum Nutr Diet*. 2012;25(2):180-188.

45. Stead M, Eadie D, McKell J, Sparks L, MacGregor A, Anderson AS. Making hospital shops healthier: evaluating the implementation of a mandatory standard for limiting food products and promotions in hospital retail outlets. *BMC Public Health*. 2020;20:132.

46. Shipton D. *Evaluation of the Implementation and Impact of the Healthcare Retail Standard in Scottish Hospitals and Other NHS Facilities in 2017*. Edinburgh, Scotland: NHS Health Scotland; 2019:140. http://www.healthscotland.scot/media/2326/evaluation-of-the-healthcare-retail-standard.pdf.

47. Welsh Assembly Government. *Review of the Implementation of the Health Promoting Hospital Vending Guidance*.; 2009. http://www.wales.nhs.uk/sites3/Documents/740/Health%20Promoting%20Hospital%20Vending%20Guidance%20Report.pdf.

48. Gray S, Orme J, Pitt H, Jones M. Food for Life: evaluation of the impact of the Hospital Food Programme in England using a case study approach. *JRSM Open*. 2017;8(10):1-9.

49. Beer-Borst S, Luta X, Hayoz S, et al. Study design and baseline characteristics of a combined educational and environmental intervention trial to lower sodium intake in Swiss employees. *BMC Public Health*. 2018;18:421.

50. Beer-Borst S, Hayoz S, Eisenblätter J, et al. RE-AIM evaluation of a one-year trial of a combined educational and environmental workplace intervention to lower salt intake in Switzerland. *Prev Med Rep*. 2019;16:100982.

51. Beer-Borst S, Eisenblätter J, Jent S, Siegenthaler S, Hayoz S. Corporate health management: evaluation of an educational and environmental intervention to promote a balanced, less salty diet. Part 2: coaching of catering teams. *Ernahrungs Umschau*. 2020;67(1):2-10.

52. Lassen A, Thorsen AV, Trolle E, Elsig M, Ovesen L. Successful strategies to increase the consumption of fruits and vegetables: results from the Danish "6 a day" Work-site Canteen Model Study. *Public Health Nutr*. 2004;7(2):263-270.

53. Young L, Bidois A, Mackay S. Making a healthy difference to menus: evaluation of a catering program in New Zealand. *Nutr Diet*. 2004;61(4):226-233.
